# Hsa_circ_0009092/miR-665/NLK signaling axis suppresses colorectal cancer progression via recruiting TAMs in the tumor microenvironment

**DOI:** 10.1186/s13046-023-02887-8

**Published:** 2023-11-27

**Authors:** Jialin Song, Qing Liu, Lei Han, Tiantian Song, Sihao Huang, Xinyao Zhang, Qiuming He, Chenxi Liang, Shuai Zhu, Bin Xiong

**Affiliations:** 1 Department of Gastrointestinal Surgery, Zhongnan Hospital of Wuhan University, Wuhan, 430071 China; 2 Hubei Key Laboratory of Tumour Biological Behaviours, Wuhan, 430071 China; 3 Department of Respiratory and Critical Care Medicine, Zhongnan Hospital of Wuhan University, Wuhan, China; 4 Wuhan Research Center for Infectious Diseases and Cancer, Chinese Academy of Medical Sciences, Wuhan, China; 5 Department of Pancreatic Surgery, Xiangya Hospital, Central South University, Changsha, 410008 Hunan China; 6 Department of General Surgery, Xiangya Hospital, Central South University, Changsha, 410008 Hunan China; 7 National Clinical Research Center for Geriatric Disorders, Xiangya Hospital, Central South University, Changsha, 410008 Hunan China

**Keywords:** Colorectal cancer, circ_0009092, miR-665, NLK, CCL2, TAMs

## Abstract

**Background:**

It has been demonstrated that circularRNA (circRNAs) plays a critical role in various cancers. While the potential molecular mechanism of circRNAs in the progression of colorectal cancer (CRC) remains uncertain.

**Methods:**

Differentially expressed circRNAs were identified by RNA sequencing. RT-qPCR detected the expression of circ_0009092, miR-665, and NLK in CRC tissues and cells. Functions of circ_0009092 on tumor cell proliferation, migration, and invasion were investigated by a series of in vitro assays. The underlying mechanism of circ_0009092 was explored by bioinformatics analysis, RNA immunoprecipitation (RIP) and luciferase assays. A co-culture assay in vitro was performed to detect the affection of circ_0009092 on macrophage recruitment in the tumor microenvironment (TME). A xenograft mouse model was used to explore the effect of circ_0009092 on tumor growth.

**Results:**

Circ_0009092 was downregulated in CRCand predicted a good prognosis. Overexpression of circ_0009092 reduced tumor cell EMT, proliferation, migration, and invasion in vitro and in vivo. Mechanistically, circ_0009092 elevated the NLK expression via sponging miR-665 and suppressed the Wnt/β-catenin signaling pathway. EIF4EA3 induced circ_0009092 expression in CRC cells. In addition, NLK regulates phosphorylation and O-GlcNAcylation of STAT3 by binding to STAT3, thereby inhibiting CCL2 expression, in which it inhibits macrophage recruitment in the tumor microenvironment (TME).

**Conclusion:**

EIF4A3 suppressed circ_0009092 biogenesis, whichinhibits CRC progression by sponging miR-665 to downregulate NLK. Circ_0009092/miR-665/NLK suppressed tumor EMT, proliferation, migration, and invasion by acting on the Wnt/β-catenin signaling pathway. NLK directly interacted with STAT3 and decreased the CCL2 expression, inhibiting the recruitment of tumor-associated macrophages (TAMs) in the TME. Our study provided novel insights into the roles of circ_0009092 as a novel promising prognostic and therapeutic target in CRC.

**Supplementary Information:**

The online version contains supplementary material available at10.1186/s13046-023-02887-8.

## Introduction

Colorectal cancer (CRC) is one of the most prevalentcancers worldwide . Despite the improvement of diagnosis and therapy, the prognosis of CRC patients remains poor [1]. Metastasis is the overwhelming cause of death in CRC patients. CRC patients with advanced stage are typically accompanied by tumor metastasis, and the five-year survival rate is very low [2, 3]. Therefore, it is urgent to explore the pathogenesis of CRC and the unknown molecular mechanism involved in tumor metastasis.

In recent years, increasing evidence have demonstrated that the tumor microenvironment (TME) plays a crucial role in tumor metastasis, which contains a variety of components, including fibroblasts, blood vessels, extracellular matrix (ECM) and immune cells [4]. Tumor‐associated macrophages (TAMs) are the dominant cell population of the TME and have a key role in tumor metastasis [5]. TAMs derive from circulating monocytes and are directed into the tumor by chemokines such as CCL5, CCL2, and CCL17 [6, 7]. Increased TAMs infiltration is markedly associated with tumor metastasis in various cancers [8, 9]. TAMs are mainly categorized into classically activated macrophages (M1 phenotype) and alternatively activated macrophages (M2 phenotype) [10]. M1-like TAMs activate anti-tumor immune response, resulting in tumor suppression. Whereas M2-like TAMs exert anti-inflammatory activities and play a crucial role in promoting tumor progression and metastasis [11, 12]. As TAMs play a central role in tumor metastasis, exploration of the unknown molecular mechanisms underlying TAMs and cancer cell interaction is essential for understanding the metastatic process in CRC.

Noncoding RNAs play critical roles in the development of malignant tumors [13, 14]. Circular RNAs (circRNAs) are important members of the noncoding RNA molecules along with the miRNAs and LncRNAs, which are characterized by the covalently closed loop structures and the absence of 3′ and 5′ ends [13, 15]. Numerous studies have demonstrated that dysregulated expression of circRNAs is associated with the pathogeneses of distinct cancer [16, 17]. An increasing number studies reported that circRNAs play key roles in tumor progression and metastasis through diverse mechanisms, including miRNA sponges, RNA-binding protein (RBP) interactions, gene transcription and translation regulators [13, 18], and the growing number of studies suggest that circRNAs may be novel potential biomarkers and therapeutic targets in CRC [19, 20]. Moreover, compelling experimental evidence shows that circRNAs regulate various cells and factors of the TME [21–23]. However, the identities of pivotal circRNAs and their functions, as well as the underlying mechanisms of the crosstalk between CRC cells and TAMs are still largely enigmatic.

In the current study, we conducted circRNA sequencing in the CRC tissue samples and identified a novel circRNA named hsa_circ_0009092, as a suppressor of CRC progression. We found that circ_0009092 is significantly downregulated in CRC tissue samples and cell lines, and its expression is positively correlated with prognosis of CRC. Then, function experiments were conducted to reveal the biological roles of circ_0009092 in tumor proliferation and metastasis. In addition, we found that circ_0009092 acts as a sponge of miR-665, thereby releasing the inhibition of NLK by miR-665 and suppressing the Wnt/β-catenin signaling pathway to inhibit epithelial mesenchymal transformation (EMT) of CRC cells. Moreover, NLK bound to the STAT3 to suppress transcriptional activation of downstream gene CCL2, leading to inhibit the recruitment of macrophage and suppress progression of CRC. Our findings illustrate the new mechanism of tumor progression and metastasis, and show new insights for the crosstalk between tumor cells and TME, provide promising therapeutic targets for CRC.

## Result

### CircRNA_0009092 was decreased in CRC tissues and cell lines

**Fig. 1:**
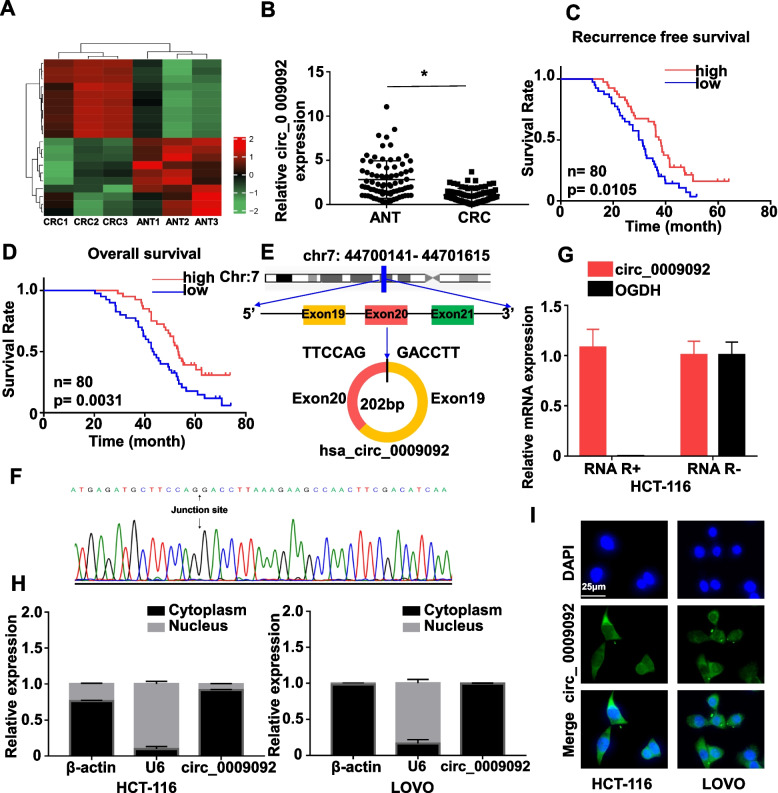
CircRNA_0009092 was decreased in CRC tissues and was a stable circRNA in CRC cells.**A**Heatmap of differentially expressed circRNA based on RNA-seq analysis in 3paired CRC tissues. The red and blue strips represent high and low expression, respectively.**B**qRT-PCR assay showing the expression level of circ_0009092 in the tumor tissue and normal tissues of CRC (*n*= 80).**C**Kaplan-Meier analysis of correlations between circ_0009092 expression levels and recurrence free survival (RFS) of 80 CRC patients.**D**Kaplan-Meier analysis of correlations between circ_0009092 expression levels and overall survival (OS) of 80 CRC patients.**E**Schematic illustration showing the OGDH exon 19-20 circularization forming circ_0009092.**F**The back-splice junction sequences of circ_0009092 were confirmed by Sanger sequencing.**G**Stability of circ_0009092 and OGDH mRNA was assessed by RNase R treatment followed by qRT-PCR in HCT116 and Lovo cells.**H**The expression levels of circ_0009092 in the nuclear and cytoplasmic fractions of HCT-116 and Lovo cells were assessed qRT-PCR.**I**FISH detection of circ_0009092 in HCT-116 and Lovo cells. Nuclei were stained with DAPI. Scale bar, 50 μm. Statistical analysis between two groups was performed using two-tailed t-test. One-way ANOVA statistical tests were adopted for more than two groups. Data are the means ± SD of three experiments. *P<0.05

In order to identify the circRNA expression profile in CRC, the circRNA microarray analysis was performed to identify the aberrant circRNA expression in three paired CRC tissues and adjacent normal tissues (ANT). The results demonstrated that 20 circRNAs were differentially expressed, including 10 upregulated and 10 downregulated circRNAs. The heatmap displayed the most upregulated and downregulated circRNAs (Fig.1A). In the present study, we selected circ_0009092 as our protagonist. Firstly, the expression level of circ_0009092 in 80 paired CRC tissues and ANT was determined. As shown in Fig.1B, the expression level of circ_0009092 was significantly downregulated in CRC tissues. Notably, circ_0009092 expression was correlated with TNM stage, lymph node involvement and metastasis (Table S1). Next, we investigated the correlation between circ_0009092 expression and the prognosis of CRC patients. Kaplan–Meier survival analysis showed that a lower level of circ_0009092 expression was associated with poor overall survival (OS) and recurrence-freesurvival (RFS) (Fig.1C-D). Based on the above results, we postulated that circ_0009092 is a key circRNA involved in CRC progression.

CircRNAs are characterized by a covalently closed-loop structure that is generated by back-splicing, eventually leading to increased structural stability. To investigate these properties of circ_0009092, we first determined the circ_0009092 expression in CRC cells and normal cells. Results showed that circ_0009092 was highly expressed in Lovo cells and expressed at low levels in HCT-116 cells, then we chose Lovo and HCT-116 cell lines for subsequent experiments (Fig. S1A). Sanger sequencing and RNase R treatment were used in HCT-116 and Lovo cells. According to circbase, circ_0009092 was derived from exons 19 and 20 of OGDH gene with a length of 202 nucleotides (Fig.1E). The presence of head-to-tail back-splicing junction in the circ_0009092 was confirmed by Sanger sequencing in the qPCR product of circ_0009092 (Fig.1F). We then treated tumor cells with RNase R (a degrader of the linear RNA) and observed that circ_0009092 was resistant to RNase R digestion compared with the linear OGDH mRNA, suggesting that circ_0009092 was more stable than liner OGDH mRNA (Fig.1G, Fig. S1B). In order to certify circ_0009092 was a circRNA, we designed two sets of primers in qPCR: divergent primers, amplifying circular form, and convergent primers, amplifying linear form. Results showed that circ_0009092 could be amplified by divergent primers in complementary DNA (cDNA) but not genomic DNA (gDNA) (Fig. S1C). We further investigated the subcellular location of circ_0009092 by nuclear/cytoplasmic fractionation and FISH assay. The results revealed that circ_0009092 was predominately expressed in the cytoplasm of CRC cells (Fig.1H-I). Taken together, these data demonstrated that circ_0009092 expression was strikingly downregulated in CRC tissues and was a stable circRNA in CRC cells.

### Circ_0009092 inhibited proliferation, migration, and invasion of CRC cells

**Fig. 2:**
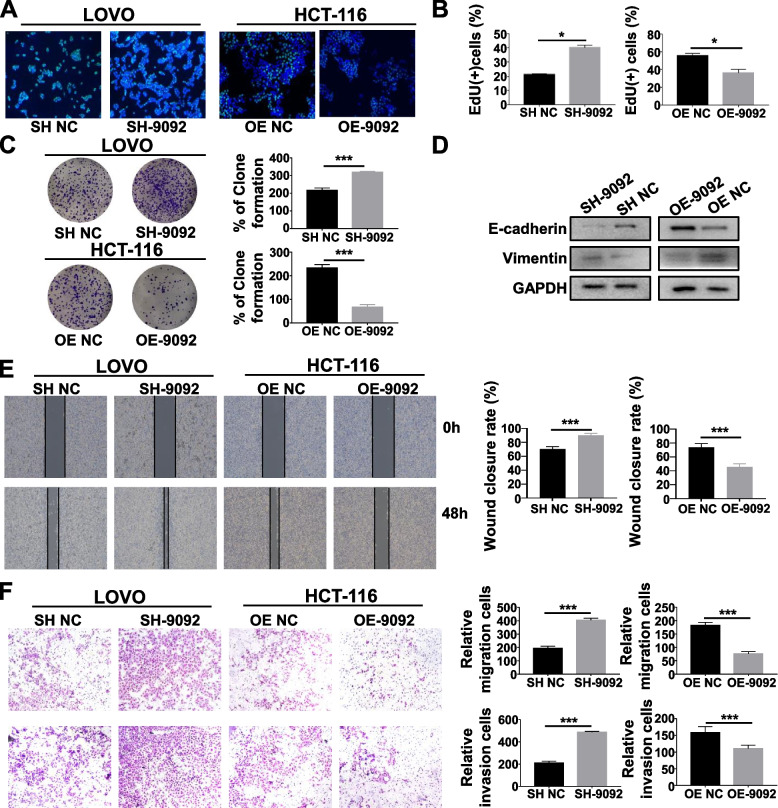
Circ_0009092 inhibited proliferation, migration, and invasion of CRC cells.**A**-**B**EdU assay was performed to detect the proliferation of Lovo and HCT-116 cells.**C**The colony formation activity of circ_0009092-depleted Lovo and circ_0009092-increased HCT-116 cells was evaluated by colony formation assay.**D**The E-cadherin and vimentin proteins in circ_0009092 transfected CRC cells as determined by Western blot.**E**-**F**Wound healing assay was used to detect the migration of circ_0009092-depleted Lovo and circ_0009092-increased HCT-116 cells.**G**-**H**The migration and invasion ability of circ_0009092-depleted Lovo and circ_0009092-increased HCT-116 cells as detected by transwell assays. Statistical analysis between two groups was performed using two-tailed t-test. One-way ANOVA statistical tests were adopted for more than two groups. Data are the means ± SD of three experiments. *P< 0.05, **P< 0.01, ***P< 0.001

Given the results that circ_0009092 expression is decreased in CRC tissues, we speculated that circ_0009092 may function as a tumor suppressor in CRC. The cell proliferation assay, the wound healing assay, and the Transwell assay with or without Matrigel were conducted to assess the effect of circ_0009092 on cell proliferation, migration, and invasion. Edu assay showed that overexpression of circ_0009092 significantly inhibited the cell proliferation of CRC cells, while knockdown of circ_0009092 markedly promoted cell proliferation (Fig.2A-B). In a clonogenic assay, overexpression of circ_0009092 lowered both the number of clones, and knockdown of circ_0009092 enhanced clonal proliferative capacity of CRC cells (Fig.2C). CircRNAs play important roles in enhancing cancer migration and invasion through regulating EMT. Therefore, the effect of circ_0009092 on the expression of EMT-related protein was determined by performing Western blot (WB) assay. WB results showed that decreased circ_0009092 significantly increased the Vimentin protein level and suppressed the expression of E-cadherin. On the contrary, there was a decreased Vimentin protein level and an increased E-cadherin expression level in the circ_0009092 overexpressed group (Fig.2D). Next, the wound healing assay showed that overexpression of circ_0009092 significantly impaired the migration of CRC cells. In the circ_0009092 knockdown group, the wound healing assay showed the contrary tendency (Fig.2E). In addition, we explored the effects of circ_0009092 on cell migration and invasion by Transwell migration and Transwell invasion assays. Results showed overexpression of circ_0009092 inhibited cell migration and invasion, while knockdown of circ_0009092 enhanced cell migration and invasion (Fig.2F). Collectively, these data demonstrated that circ_0009092 inhibited proliferation, migration, and invasion of CRC cells.

### Circ_0009092 functioned as a sponge for miR-665

**Fig. 3:**
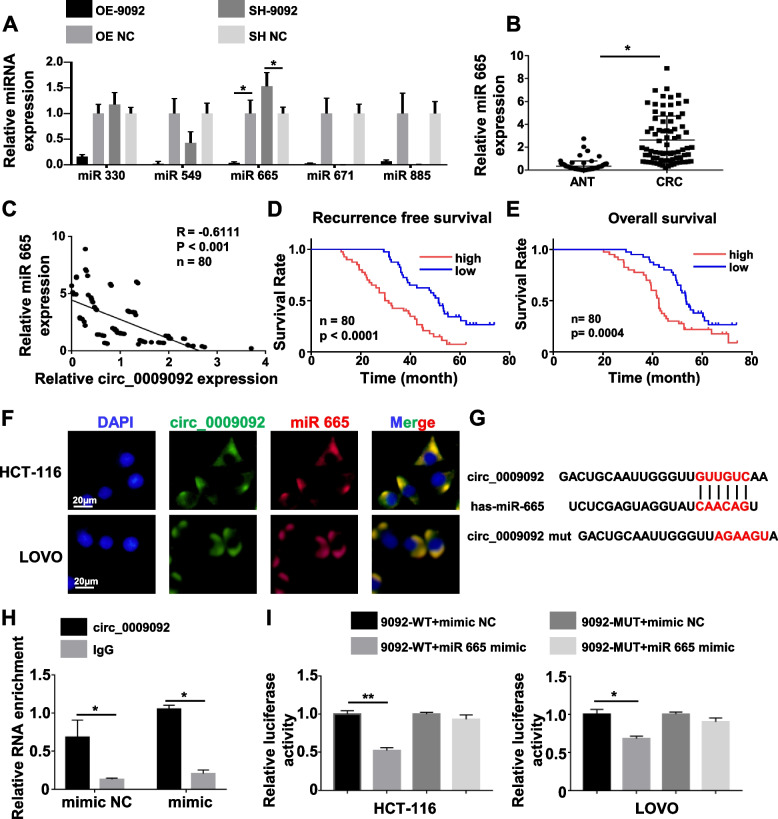
Circ_0009092 acted as the sponge of miR-665 in CRC cells.**A**qRT-PCR assay showing the expression levels of potential miRNA in the transfected CRC cells.**B**qRT-PCR analysis was performed to show the expression levels of miR-665 in 80 paired CRC tissues. (**C**) Spearman’s correlation analysis revealed a negative association between miR-665 expression and circ_0009092 expression in 80 CRC tissues.**D**Kaplan–Meier plotter of the association of circ_0009092 with RFS in 80 CRC patients.**E**Kaplan–Meier plotter of the association of circ_0009092 with OS in 80 CRC patients.**F**FISH analysis of the cellular colocalization of miR-665 and circ_0009092 in HCT-116 and Lovo cells. Scale bar, 20 μm.**G**Schematic representation of binding sequences between miR-665 and circ_0009092.**H**RIP assay was conducted for detecting the binding of circ_0009092 and miR-665 by using the Ago2 antibody.**I**The luciferase activity of the WT luc-circ_0009092 or Mut luc-circ_0009092 after transfection with miR-665 mimic in CRC cells. Statistical analysis between two groups was performed using two-tailed t-test. One-way ANOVA statistical tests were adopted for more than two groups. Data are the means ± SD of three experiments. *P< 0.05, **P< 0.01

CircRNA exerted diverse biological functions by acting as microRNA “sponges”. Therefore, we assessed whether circ_0009092 could sponge miRNA to suppress CRC progression. Five potential miRNAs (miR-330, miR-549, miR-665, miR-671, miR-885) were predicted by the Circinteractome and starBase database. We analyzed the expression levels of these five miRNAs in CRC cells and found that miR-665 was markedly correlated with circ_0009092 (Fig.3A). QPCR results indicated that the expression of miR-665 was remarkably increased by circ_0009092 knockdown, while circ_0009092 overexpression resulted in downregulation of miR-665 in CRC cells (Fig.3A). In addition, miR-665 expression levels were markedly overexpressed (Fig.3B) and negatively correlated with circ_0009092 expression in CRC tissues (Fig.3C,*R*=-0.6111,*P*< 0.001). Kaplan-Meier survival analysis showed that high expression of miR-665 was correlated with worse RFS (Fig.3D) and OS (Fig.3E) in CRC patient. In addition, the FISH assay was applied to assess the subcellular co-location of circ_0009092 and miR-665. The results demonstrated that circ_0009092 and miR-665 were colocalized in the cytoplasm of HCT-116 and Lovo cells, suggesting that circ_0009092 could interact with miR-665 and function in the cytoplasm (Fig.3F). FISH analysis of CRC samples and ANT confirmed the downregulation of circ_0009092 and upregulation of miR-665 in CRC samples compared to ANT (Fig. S2A).

We further explored whether circ_0009092 exerts its regulatory effects by sponging miR-665. Bioinformatic analysis was used to predict the potential binding sites of miR-665 on circ_0009092 (Fig.3G). We performed RNA immunoprecipitation (RIP) for argonaute 2 (AGO2) in tumor cells and investigated the expression of circ_0009092 and miR-665 by qPCR to verify the binding effect between circ_0009092 and miR-665. MiRNA transfer into AGO2 protein to form a AGO2/RNA-induced silencing complex (RISC). In RIP assay, compared with control IgG, circ_0009092 was preferentially enriched in miRNA ribonucleoprotein complexes containing AGO2, and miR-665 was highly expressed in the AGO2 pellet compared with that in the IgG control. Moreover, circ_0009092 was highly enriched in the miR-665-overexpressed (miR-665 mimic) group compared with that in the negative control (mirR-665 mimic NC) group (Fig.3H). In addition, dual-luciferase activity analysis was performed to verify that miR-665 binds to circ_0009092 in CRC cells. The sequences of circ_0009092 containing wild or mutant miR-665 binding site were cloned into the luciferase reporter vector pLG3. The results demonstrated that miR-665 mimic significantly reduces the luciferase activity of the WT circ_0009092, but had no effect on that of the MUT circ_0009092 compared with the NC group (Fig.3I). Taken together, these results indicated that the interaction of circ_0009092 and miR-665 lead to the down expression of miR-665 in CRC cells.

### Circ_0009092 inhibited CRC cell progression by targeting miR-665

**Fig. 4:**
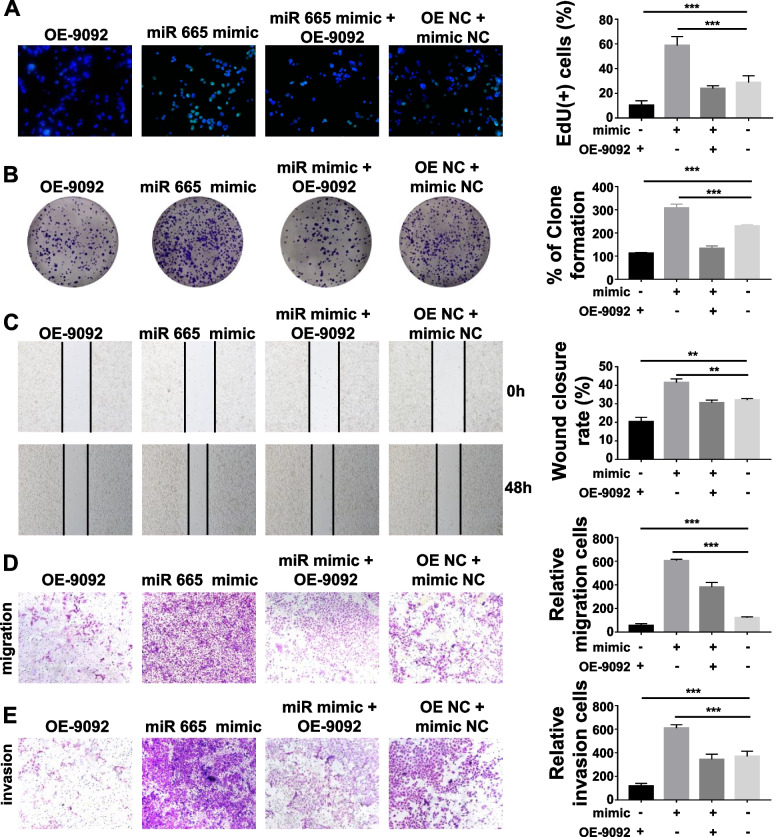
Circ_0009092 inhibited CRC cell progression by targeting miR-665.**A**EdU assays were utilized to detect the viability and proliferation of control, OE-circ_0009092, miR-665 mimic, and OE-circ_0009092+miR-665 mimic -treated HCT-116 cells.**B**The colony formation activities of control, OE-circ_0009092, miR-665 mimic, and OE-circ_0009092+miR-665 mimic -treated HCT-116 cells were evaluated.**C**Wound healing assay were used to detect the migration of control, OE-circ_0009092, miR-665 mimic, and OE-circ_0009092+miR-665 mimic -treated HCT-116 cells.**D**The migration and invasion ability of control, OE-circ_0009092, miR-665 mimic, and OE-circ_0009092+miR-665 mimic -treated HCT-116 cells were detected by transwell assays. Statistical analysis between two groups was performed using two-tailed t-test. One-way ANOVA statistical tests were adopted for more than two groups. Data are the means ± SD of three experiments. *P< 0.05, **P< 0.01, ***P< 0.001

To explore that circ_0009092 regulates tumor progression by sponging miR-665, miR-665 knockdown and overexpression cell lines were constructed. EdU (Fig.4A), colony formation (Fig.4B), wound healing (Fig.4C) and transwell assays (Fig.4D) showed that miR-665 overexpression significantly promoted cell proliferation and migration capacities of CRC cells. To further investigate the roles of circ_0009092/miR-665 in CRC progression, we performed rescue assay. EdU (Fig.4A) and colony formation assay (Fig.4B) revealed that miR-665 mimic efficiently restored the proliferative capacity of circ_0009092-overexpressed cells. The tumor-promoting effect induced by circ_0009092 knockdown was abrogated by inhibition of miR-665 (Fig. S3A-B). Similarly, wound healing (Fig.4C, Fig. S3C) and Transwell assays (Fig.4D-E, Fig. S3D-E) showed that the promotive effects on migratory and invasive abilities induced by circ_0009092 knockdown were restored by miR-665 overexpression. Together, these data indicated that circ_0009092 regulates CRC progression via sponging miR-665.

### NLK was the target of miR-665 in CRC cells

**Fig. 5:**
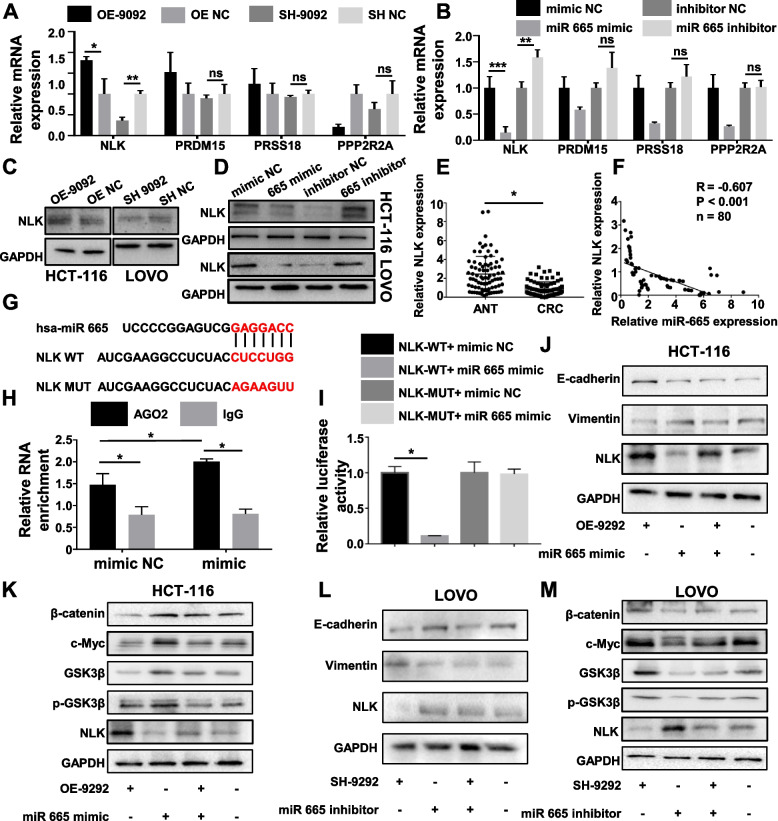
NLK functioned as the target of miR-665 in CRC cells.**A**Expression of potential target genes in CRC cells transfected with OE-circ_0009092, SH- circ_0009092. (**B**) qRT-PCR analysis of potential target genes in CRC cells transfected with miR-665 mimic, mimic NC, miR-665 inhibitor, inhibitor NC.**C**NLK protein expression was determined by Western blot in HCT116 and Lovo cells transfected with OE-circ_0009092, SH- circ_0009092.**D**NLK protein expression was determined by Western blot in HCT116 and Lovo cells transfected with miR-665 mimic, mimic NC, miR-665 inhibitor, inhibitor NC.**E**The expression of NLK in 80 paired CRC tissues was detected by qRT-PCR.**F**The correlation between NLK and miR-665 expression in 80 paired CRC tissues.**G**Schematic representation of potential binding sites between miR-665 and NLK.**H**RIP assay was conducted for detecting the binding of miR-665 and NLK by using the Ago2 antibody in CRC cells transfected with miR-665 mimic, mimic NC.**I**Relative luciferase activities were determined in CRC cells transfected with NLK 3′-UTR WT or MUT luciferase reporter vectors and miR-665 mimic or mimic NC.**J**Western blot showed the protein changes of E-cadherin, Vimentin, and NLK in control, OE-circ_0009092, miR-665 mimic, and OE-circ_0009092+miR-665 mimic -treated HCT-116 cells.**K**Western blot showed the protein changes of Wnt/β-catenin signaling pathway related proteins in control, OE-circ_0009092, miR-665 mimic, and OE-circ_0009092+miR-665 mimic-treated HCT-116 cells.**L**Western blot showed the expression changes of E-cadherin, Vimentin, and NLK in Lovo cells by treatment of SH-circ_0009092, miR-665 inhibitor, and SH-circ_0009092+miR-665 inhibitor.**M**Western blot showed the protein changes of Wnt/β-catenin signaling pathway related proteins in SH-circ_0009092, miR-665 inhibitor, and SH-circ_0009092+miR-665 inhibitor-treated Lovo cells. Statistical analysis between two groups was performed using two-tailed t-test. One-way ANOVA statistical tests were adopted for more than two groups. Data are the means ± SD of three experiments. *P< 0.05, **P< 0.01, ***P< 0.001

Based on TargetScan, miRWalk, and Starbase databases, NLK, PRDM15, PRSS18, and PPP2R2A were identified as the potential targets of miR-665. Overexpression of circ_0009092 induced the expression of NLK mRNA in HCT-116 and Lovo cells, while knockdown of circ_0009092 exerted the opposite effects. However, exogenous expression or knockdown of circ_0009092 had no effect on the expression of PRDM15, PRSS18, and PPP2R2A (Fig.5A, Fig. S4A). In addition, miR-665 mimic significantly suppressed the expression of NLK mRNA and miR-665 inhibitor markedly increased NLK mRNA expression in HCT-116 and Lovo cells (Fig.5B). Similarly, the WB results validated that NLK expression was regulated by circ_0009092 and miR-665 in CRC cells (Fig.5C-D). We further examined the expression of NLK in tumor tissues of CRC. The NLK mRNA levels were significantly lower in 80 tumor samples than that in ANT (Fig.5E). Furthermore, the expression level of NLK in CRC tissues was positively correlated with circ_0009092 expression and negatively correlated with miR-665 expression (circ_0009092*p*< 0.001, miR-665*p*< 0.001, Fig.5F, Fig. S4B). Then, the binding ability of miR-665 to NLK mRNA was detected by RIP assay using the antibody against AGO2. NLK was highly expressed in the AGO2 pellet compared with that in the IgG control. In addition, overexpression of miR-665 significantly enriched the association of NLK mRNA with the AGO2-containing complex, suggesting the interaction between miR-665 and NLK (Fig.5H). We further found that miR-665 mimics could reduce the luciferase activity of the WT NLK- 3’ -UTR, but had no effect on that of the Mut NLK-3’-UTR compared with the NC group in HCT-116 and Lovo cells (Fig.5I, Fig. S4C).

It has been reported that NLK is an effector of the Wnt/β-catenin signaling pathway, we speculated that circ_0009092/miR-665/NLK regulates the Wnt/β-catenin signaling pathway. Western blot showed that overexpression of circ_0009092 markedly attenuated the expression of EMT-related proteins (Fig.5J), β-catenin, c-myc (target of Wnt/β-catenin) and p-GSK-3β (Fig.5K), while circ_0009092 knockdown achieved an opposite result (Fig.5L-M). We also determined the expression of EMT-related proteins, β-catenin, c-myc and p-GSK-3β inhibited by overexpressed circ_0009092 was significantly increased after transfected with miR-665 mimic (Fig.5J-K). NLK knockdown reversed the miR-665 inhibitor-mediated Wnt/β-catenin signaling pathway (Fig.5L-M). Overall, these data indicated that circ_0009092 could impair the suppression of miR-665 to NLK, which further regulated the Wnt/β-catenin signaling pathway. In CRC, the down expression of circ_0009092 led to the inhibition of NLK, then the Wnt/β-catenin signaling pathway was suppressed.

### Circ_0009092/miR-665/NLK inhibited TAMs recruitment by suppressing CCL2 secretion in CRC

**Fig. 6:**
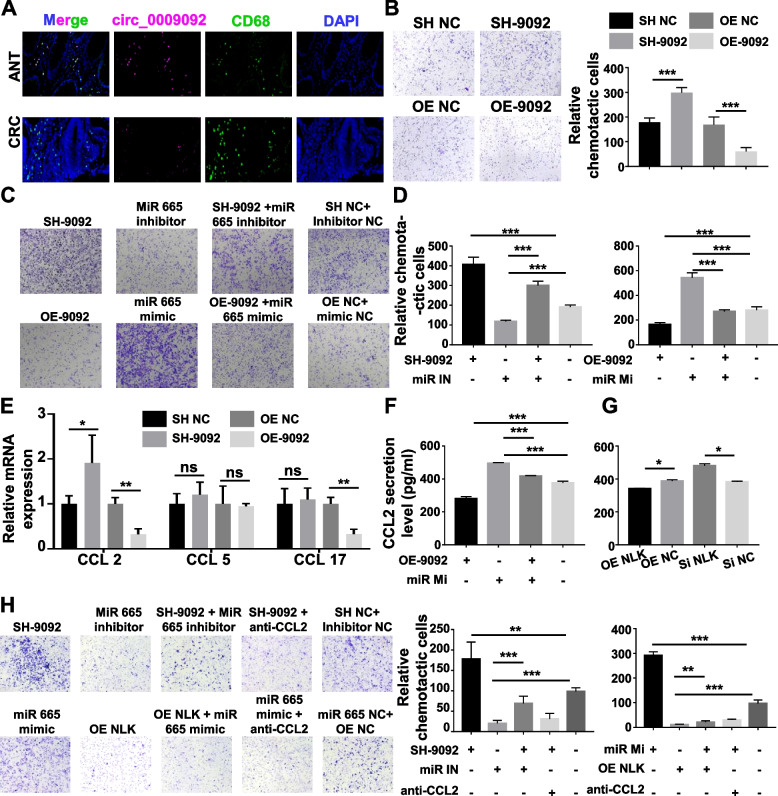
Circ_0009092 regulated the recruitment of macrophages in CRC cells.**A**Representative immunofluorescence photographs for circ_0009092, CD68, DAPI in CRC samples. Bar = 50μm.**B**Chemotaxis analysis of THP-1 macrophages toward supernatant from OE-circ_0009092, SH-circ_0009092-treated CRC cells.**C**Chemotaxis analysis of THP-1 macrophages toward supernatant from OE-circ_0009092, SH-circ_0009092, miR-665 mimic and miR-665 inhibitor.**D**Quantification analysis of chemotactic cells in five fields was counted manually.**E**The potential chemokines which promoted macrophage infiltration were identified by qRT-PCR in OE-circ_0009092, SH-circ_0009092-treated CRC cells.**F**ELISA assay detected the CCL2 secretion level in OE-circ_0009092 or miR-665 mimic-treated CRC cells.**G**ELISA assay detected the CCL2 secretion level in OE-NLK or SI-NLK-treated CRC cells.**H**Chemotaxis analysis of THP-1 macrophages toward SH-circ_0009092 CRC cell supernatant with or without CCL2 Ab, miR-665 mimic CRC supernatant with or without CCL2 Ab.**I**Quantification analysis of chemotactic cells in five fields was counted manually. Statistical analysis between two groups was performed using two-tailed t-test. One-way ANOVA statistical tests were adopted for more than two groups. Data are the means ± SD of three experiments. *P< 0.05, **P< 0.01, ***P< 0.001

Since the infiltration of immune cells in the TME is highly relevant to the CRC progression, we analyzed the distribution of different immune cells in CRC tissues and normal tissues though the Bioinformatics algorithm. We integrated six latest algorithms, including TIMER, xCell, MCP-counter, CIBERSORT, EPIC and quanTIseq. Results showed that The CRC tumors displayed massive macrophage infiltration, especially M2 macrophage infiltration (Fig. S5A-B). In addition, circ_0009092 was found to be inversely correlated with the density of TAMs marker CD68 in CRC (Fig.6A). These observations led us to speculate that circ_0009092 was correlated with TAMs infiltration. An in vitro model of TAMs was then applied. THP-1 was treated with Phorbol 12-myristate 13-acetate (PMA) and co-cultured with tumor cells for 48h. We observed that THP-1 macrophages were recruited by circ_0009092 knockdown, and overexpression of circ_0009092 inhibited the recruitment of macrophages (Fig.6B). In addition, miR-665 mimic markedly promoted the recruitment of macrophages, and the miR-665 inhibitor could restrain the recruitment of macrophages induced by circ_0009092 knockdown (Fig.6C-D). In addition, overexpression of NLK (oe-NLK) failed to recruit macrophages and knock down of NLK (si-NLK) showed the opposite effect. MiR-665 inhibitor substantially inhibited si-NLK-induced the recruitment of macrophages. While miR-665 mimic recovered the effect of oe-NLK (Fig. S6A-B). Thus, our results substantiated that circ_0009092/miR-665/NLK inhibits TAMs recruitment in the TME of CRC.

We then analyzed the potential chemokines associated with TAMs recruitment. RT-PCR results showed that mRNA expression of CCL2 emerged as the most prominently chemokine in circ_0009092 transfected tumor cells (Fig.6E). ELISA analysis showed that CCL2 protein levels were significantly increased in the media from miR-665 mimic, sh- circ_0009092, or si-NLK group compared to NC group (Fig.6F-G). Rescue experiments suggested that miR-665 mimic-induced CCL2 could be reversed by circ_0009092 overexpression (Fig.6F, Fig. S6C). In addition, CCL2 neutralizing antibody suppressed the circ_0009092 knockdown-enhanced the recruitment of macrophages (Fig.6H). Collectively, these data suggest that circ_0009092/miR-665/NLK inhibits the recruitment of TAMs via CCL2.

### NLK bound to STAT3 protein and regulated its activity to suppress CCL2 expression

**Fig. 7:**
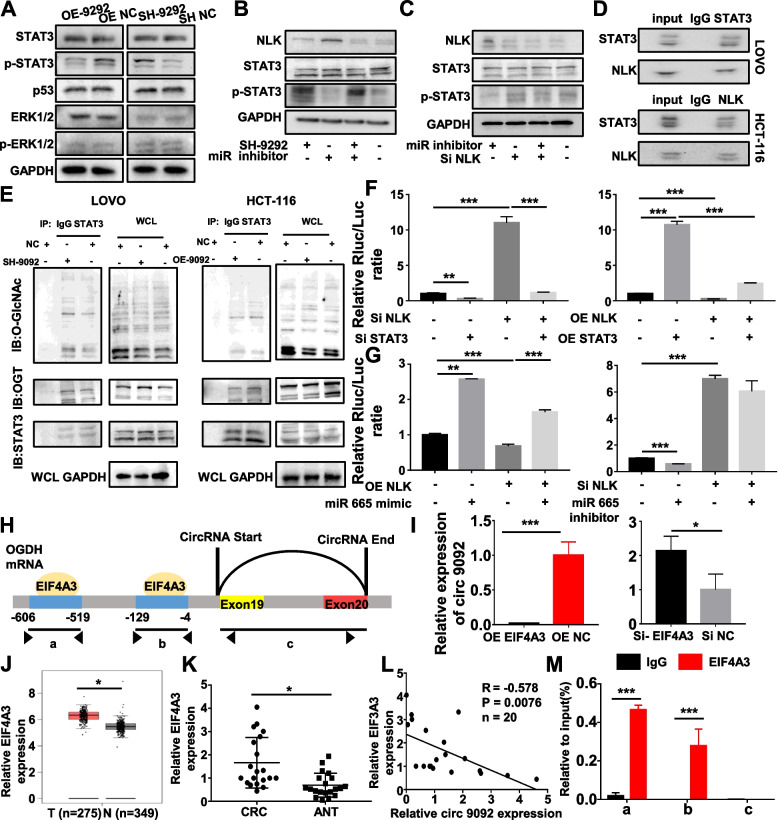
NLK regulated the STAT3 and EIF4A3 accelerated the biogenesis of circ_0009092 in CRC cells.**A**Western blot analysis of p53, STAT3, ERK1/2 signaling pathway in CRC cells transfected with OE-circ_0009092 or SH-circ_0009092.**B**Western blot analysis of CRC cells transfected with SH-circ_0009092, miR-665 inhibitor, and SH-circ_0009092+miR-665 inhibitor.**C**Western blot analysis of CRC cells transfected with SI-NLK, miR-665 inhibitor, and SI-NLK+miR-665 inhibitor.**D**CoIP experiment of endogenous NLK and STAT3 in Lovo and HCT-116 cells.**E**Interaction between STAT3 and OGT/O-GlcNAcylation modification was evaluated by coIP assay in CRC cells transfected with OE-circ_0009092 or SH-circ_0009092, WCL indicates whole cell lysates.**F**-**G**The relative CCL2 promoter transcriptional activity was detected in NLK, STAT3, miR-665 transfected CRC cells by dual luciferase assay and normalized to Renilla luciferase activity.**H**The putative binding sites of EIF4A3 in the upstream region of the circ_0009092 predicted with circinteractome database.**I**qRT-PCR analysis of circ_0009092 expression in EIF4A3-overexpressed and EIF4A3-knockdown CRC cells.**J**GEPIA database was used to evaluate the expression of EIF4A3 in 275 tumor tissues and 349 normal tissues of CRC.**K**qRT-PCR analysis of EIF4A3 in 20 paired CRC tissues.**L**The correlation between EIF4A3 and circ_0009092 expression in 20 paired CRC tissues.**M**RIP assay analysis of interaction of EIF4A3 with circ_0009092 in CRC cells. IgG was used as negative control. Statistical analysis between two groups was performed using two-tailed t-test. One-way ANOVA statistical tests were adopted for more than two groups. Data are the means ± SD of three experiments. *P< 0.05, **P< 0.01, ***P< 0.001

To explore the molecular mechanism by which circ_0009092/miR-665/NLK regulated CCL2, we searched for ERK1/2, STAT3 and p53 signaling pathways, of which all had been reported to be the major molecule involved in the regulation of CCL2. WB results showed that p-STAT3 (Tyr705) significantly increased after circ_0009092/miR-665/NLK treatment, whereas there was no significant change in the phosphorylation level of ERK1/2 (Fig.7A). Rescue experiments showed that miR-665 inhibitor could not significantly change the expression of p-STAT3 after circ_0009092 knockdown (Fig.7B). Furthermore, si-NLK markedly alter miR-665 inhibitor-inhibited p-STAT3 expression (Fig.7C). These results suggested that the circ_0009092/miR-665/NLK signal can regulate the p-STAT3 expression. It has been reported that NLK could bind to a wide range of transcription factors and regulated the transcriptional activity. We assessed whether NLK was capable of binding to STAT3, thus affecting CCL2 expression. We carried out a co-immunoprecipitation (CoIP) assay in tumor cells using the antibody against STAT3 or NLK. The CoIP results demonstrated the binding of NLK with STAT3 (Fig.7D). In addition, IF results showed the co-localization of NLK and STAT3 in HCT-116 and Lovo cells (Fig. S7A). Besides of phosphorylation, STAT3 protein can also be modified by O-GlcNAcylation. O-GlcNAcylation is a key posttranslational modification, a single monosaccharide, β-O-GlcNAc is added onto serine or threonine residues of proteins. The O-GlcNAcylation occurs in an analogous fashion to protein phosphorylation, and there is an extensive crosstalk between O-GlcNAcylation and phosphorylation [24]. Previous studies showed that O-GlcNAcylation of STAT3 could promote STAT3 phosphorylation [25], so we wondered whether circ_0009092/miR-665/NLK could affect O-GlcNAcylation of STAT3. CoIP assay was performed and revealed that knockdown of circ_0009092 dramatically increased the O-GlcNAcylation modification level of STAT3, and increased binding between O-linked N-acetylglucosamine transferase (OGT) and STAT3. Meanwhile, overexpression of circ_0009092 reduced STAT3 O-GlcNAcylation modification and conjunction with OGT (Fig.7E). In addition, miR-665 inhibitor inhibited the STAT3 O-GlcNAcylation modification and conjunction with OGT (Fig. S7B), while miR-665 mimic increased the STAT3 O-GlcNAcylation modification and conjunction with OGT (Fig. S7C). These results demonstrated that circ_0009092/miR-665/NLK regulate STAT3 by binding with STAT3 and regulating its phosphorylation and O-GlcNAcylation. Then we determined STAT3 transcriptional activity by a promoter reporter assay in which luciferase reporter was driven by a CCL2 promoter. The results showed that STAT3 induced the promoter activity of CCL2 in tumor cells transfected with the promoter region of CCL2. Luciferase activity of CCL2 promoter was significantly decreased in transfected with knockdown of STAT3 (Fig.7F). Meanwhile, si-NLK significantly increased the STAT3-mediated transcription activity, and oe-NLK inhibited STAT3-mediated transcription activity of CCL2 promoter. MiR-665 mimic could increase the luciferase activity of CCL2 promoter that was restrained by oe-NLK, and miR-665 inhibitor could not reverse the luciferase activity of CCL2 promoter that was increased by si-NLK (Fig.7G). Moreover, overexpression of circ_0009092 reversed the luciferase activity of CCL2 promoter that was increased by miR-665 mimic (Fig. S7D), and knockdown of circ_0009092 increased the luciferase activity of CCL2 promoter that was reduced by miR-665 inhibitor (Fig. S7E). Taken together, these results demonstrate that NLK bound to STAT3 and increases its transcriptional activity of CCL2.

### EIF4A3 suppressed circ_0009092 expression in CRC cells

RNA-binding proteins (RBPs), such as QKI, ESRP1, and EIFEA3, can drive cyclization of RNA to regulate the biogenesis of circRNAs. Therefore, CircInteractome was used to predict the RNA binding proteins that match the flanking regions of the circ_0009092, of which EIF4A3 has the most binding sites (Fig.7H). PCR results showed that the expression of circ_0009092 was significantly decreased after the overexpression of EIF4A3. Knockdown of EIF4A3 markedly increased the expression of circ_0009092 in CRC cells (Fig.7I). GEPIA was used to examine the expression of EIF4A3 in CRC samples, and results showed that EIF4A3 expression was increased in CRC tissues than in the ANT (Fig.7J). In addition, qPCR results also revealed that the expression of EIF4A3 was elevated in CRC tissues than in the ANT (Fig.7K). And the expression level of EIF4A3 mRNA was negatively correlated with circ_0009092 expression level, suggesting that EIF4A3 is related to circ_0009092 expression (Fig.7L). In addition, the RIP assay using anti-EIF4A3 antibody showed that EIF4A3 bound with circ_0009092 through two upstream binding sites a and b but not circ_0009092 (region c) in the RNA-protein complex (Fig.7M). Taken together, these data demonstrated that circ_0009092 is regulated by EIF4A3 and EIF4A3 suppresses circ_0009092 expression by binding to flanking sequences.

### Circ_0009092 hampered the progression of colorectal cancer in vivo

**Fig. 8:**
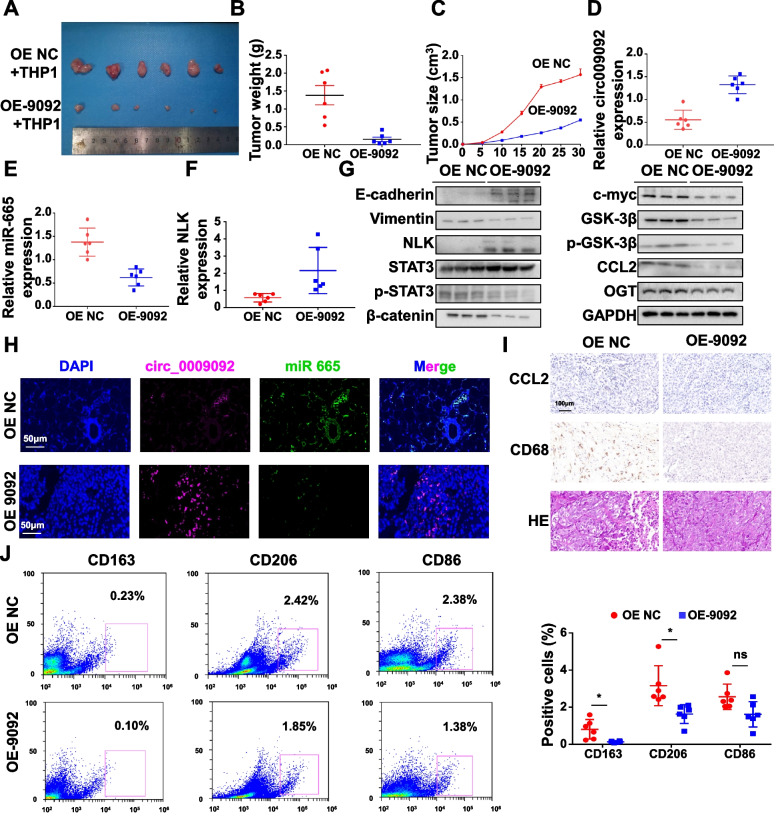
Circ_0009092 inhibited the CRC tumor growth in vivo.**A**Images of xenograft tumors formed by HCT-116 cells stably expressing OE-circ_0009092, OE-NC and THP1 cells.**B**The tumor weights of xenografts were evaluated.**C**The tumor growth curves of the OE-circ_0009092 and the OE-NC group was shown.**D**qRT-PCR analysis of circ_0009092 expression in the OE-circ_0009092 and the OE-NC group.**E**qRT-PCR analysis of miR-665 expression in the OE-circ_0009092 and the OE-NC group.**F**qRT-PCR analysis of NLK expression in the OE-circ_0009092 and the OE-NC group.**G**WB analysis of E-cadherin, Vimentin, NLK, STAT3, p- STAT3, β-catenin, GSK3β, p-GSK3β, c-myc, and CCL2 in the OE-circ_0009092 and the OE-NC group.**H**FISH analysis of the cellular colocalization of miR-665 and circ_0009092 in the OE-circ_0009092 and the OE-NC group. Scale bar, 50 μm.**I**immunohistochemistry staining (IHC) and H&E staining of xenograft tumors.**J**Flow cytometry for analyzing the expression of M2 marker (CD163, CD206) and M1 marker (CD86) on macrophages in the tumor.*n*= 6/group. Statistical analysis between two groups was performed using two-tailed t-test. One-way ANOVA statistical tests were adopted for more than two groups. Data are the means ± SD of three experiments. *P< 0.05, **P< 0.01, ***P< 0.001. ns: no significance

**Fig. 9:**
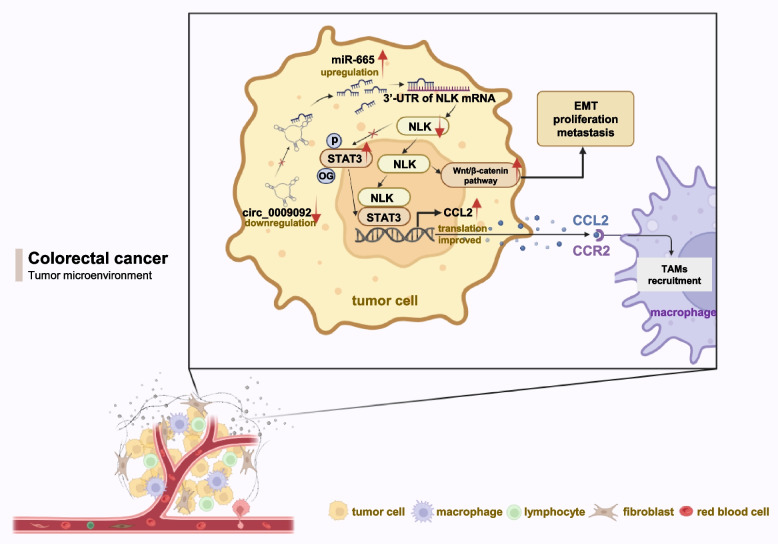
Schematic illustration indicates the mechanism by which circ_0009092/miR-665/NLK axis aggravates CRC progression and affects the recruitment of macrophages in the TME

To evaluate the function of circ_0009092 in vivo, HCT-116 cells with stably expressing either negative control (OE-NC) or OE-circ_0009092 were subcutaneously injected into the BALB/c nude mice, and THP-1 cells were injected into the caudal vein of mice. It was found that the circ_0009092 overexpression group showed significantly lower tumor weights and volumes compared to the NC group (Fig.8A-C). Circ_0009092 overexpression group showed a lower expression of miR-665 and higher expression of NLK compared with the OE-NC group (Fig.8D-F). WB results showed that circ_0009092 affects the expression of NLK, CCL2, p-STAT3, OGT, and Wnt/β-catenin singaling pathway related protein (Fig.8G). FISH analysis showed the same trend as the PCR and WB results (Fig.8H). Additionally, decreased CCL2 level and CD68 (the macrophage marker) expression level were detected in immunohistochemistry (IHC) staining in the circ_0009092 overexpression group (Fig.8I), which indicated that the circ_0009092 overexpression group presented less macrophage infiltration compared to OE-NC group. Tumors of OE-circ_0009092 group exhibited markedly reduced recruitment of macrophages as compared with OE-NC group (Fig.8J). Furthermore, the number of M2 macrophages (CD163, CD206) was decreased in the OE-circ_0009092 group compared with OE-NC group, while the number of M1 macrophages (CD86) showed no significant difference between two groups (Fig.8J). Therefore, these results corroborated that circ_0009092/miR-665/NLK inhibits CRC growth and macrophage recruitment in vivo. Taken together, our data demonstrated that circ_0009092/miR-665/NLK suppresses tumor progression and TAMs recruitment by inhibiting the Wnt/β-catenin signaling pathway in the CRC (Fig.9).

## Discussion

CircRNAs, as non-coding RNA molecules, are highly stable molecules which generated in a spliceosome-dependent process known as back-splicing. Recently, there is growing evidence that circRNAs have key roles in cancer development and progression. In the present study, we first identified that circ_0009092 was observably decreased in CRC tissues. The correlative analyses between the clinical pathological characteristics and circ_0009092 expression showed that circ_0009092 was significantly correlated with CRC differentiation, The univariate and multivariate analysis demonstrated in CRC, circ_0009092 was an independent prognostic factor for survival. The CRC is classified into different pathological molecular subtypes. Further investigation of the association between circ_0009092 and CRC grade, molecular subtype, and degree of malignancy is required to deeply understand the potential biomarker role of circ_0009092 in CRC. Functionally, we found that the circ_0009092 significantly suppressed cell proliferation, migration, and invasion, as well as inhibited CRC oncogenesis in vivo. Moreover, circ_0009092 inhibited the recruitment of TAMs in the vitro and in vivo. Mechanistically, we found that circ_0009092 functions as a sponge for miR-665 to regulate NLK expression, which bound to STAT3 to restrain the CCL2 expression, thereby leading to inhibit the EMT, the recruitment of TAMs, invasion, and metastasis of CRC. Our study indicates that circ_0009092 plays a vital role in tumorigenesis and metastasis of CRC, and it may serve as a novel diagnostic and prognostic marker for CRC.

Accumulating evidence showed that circRNAs in the cytoplasm can act as miRNA sponges to induce the dysregulated function of miRNAs and target genes. For instance, Xu et.al found that circRNA_0000392 promotes CRC progression through the miR-193a-5p/PIK3-AKT signaling pathway [26]; circPACRGL promotes progression of CRC via miR-142-3p/miR-506-3p [27]; CircALG1 promotes the progression and metastasis of CRC by binding to miR-342-5p [28]; CircHERC4 promotes tumor metastasis through absorbing miR-556-5p in CRC [29]. Here, we reported the role of circ_0009092 as miRNA sponge. Through bioinformatic analyses, we found that miR-665 is potentially regulated by circ_0009092, and the combination of circ_0009092 and miR-665 was verified by RIP assay and luciferase reporter experiment. Furthermore, rescue experiments showed that the suppression induced by circ_0009092 on the cell proliferation, migration and invasion can be reversed by miR-665. Collectively, our results demonstrated that circ_0009092 acts as the sponge of miR-665 to participate in the proliferation and metastasis of CRC.

MicroRNAs, as non-coding RNAs, play vital roles in tumor progression and metastasis through modulating EMT. In various cancer, miRNAs were found to be the crucial approach in EMT, so the circRNAs could regulate EMT by targeting EMT-related miRNAs. Recent studies have shown conflicting results that miR-665 was involved in tumor EMT as oncogene or tumor suppressor. MiR-665 has been identified as an oncogene in breast cancer [30], gastric cancer [31], and hepatocellular cancer [32], whereas acted as tumor suppressor gene in bladder cancer [33], cervical cancer [34], and pancreatic cancer [35]. While the effects of miR-665 in CRC progression is less clear. In the present study, we found that miR-665 could promote CRC invasion and metastasis by promoting the EMT process. As noncoding RNAs, miRNAs exert biological effects by modulating their target genes. Based on bioinformatic analysis and experimental validation, we identified that NLK was the target of miR-665. We further confirmed that circ_0009092 could bind to miR-665, leading to diminish the repression of miR-665 on NLK 3′-UTR and eventually suppressed CRC progression.

The Wnt/β-catenin signaling pathway comprises a family of proteins that play critical roles in embryonic development and adult tissue homeostasis [36, 37]. The aberrant activation of Wnt/β-catenin signaling often leads to tumor EMT in various cancers including CRC [38, 39]. NLK is a member of the mitogen-activated protein kinase family that regulates a wide range of transcription factors [40], and it is considered a tumor suppressor targeting Wnt/β-catenin signaling pathway in diverse cancers, such as ovarian cancer [41], glioma [42], and hepatocellular cancer [43]. Consistently, our results showed that the expression level of NLK was decreased in CRC tissues than that in ANT, and the low NLK expression was significantly correlated with shorter survival in CRC. We then explored whether circ_0009092/miR-665/NLK affected Wnt/β-catenin signaling pathway. Our results showed the related genes of Wnt/β-catenin signaling pathway, such as β-catenin, GSK-3β, c-myc, and EMT-related proteins were affected by circ_0009092/miR-665/NLK, indicating circ_0009092/miR-665/NLK inhibits Wnt/β-catenin signaling pathway and suppresses EMT progression in CRC.

TAMs are the most abundant type of infiltrating immune cells in the TME. Recent studies suggest that noncoding RNAs, such as miRNA and lncRNA, are involved in the recruitment of TAMs via exosomes, chemokines, and cytokines. While little is known about the regulatory mechanism of circRNA and TAMs in CRC. Our findings offer a novel angle that circ_0009092 regulates CCL2 to affect TAMs recruitments. STAT3 is a DNA-binding factor that regulates CCL2 via its binding activity [44]. It is well known that STAT3 exerts its biological function largely depending on its phosphorylation at Ser727 and Tyr705 [45]. Recent studies have identified additional posttranslational modifications on STAT3 other than phosphorylation, including O-GlcNAcylated STAT3, and the relationship has been reported previously between O-GlcNAcylation and phosphorylation of STAT3 [25, 46]. As an important posttranslational modification, O-GlcNAcylation subtly modulates its target proteins’ function [47]. It was reported Myeloid-derived cullin 3 enhanced STAT3 phosphorylation and transcriptional activity by inhibiting O-GlcNAcylation during intestinal inflammation [46]. While OGT-mediated O-GlcNAcylation promoted STAT3 phosphorylation and transcriptional activity in lung cancer [25]. The protein O-GlcNAcylation and phosphorylation modifications are dynamically regulated to coordinate the pathological environment. Based on the cross-regulation between O-GlcNAcylation and phosphorylation. Our data demonstrate for the first time that NLK can bind to STAT3 and regulate its O-GlcNAcylation and phosphorylation, thereby affecting its translational activity, which is critical for the secretion of CCL2. Moreover, there is extensive evidence that TAMs enhance tumor development and metastasis by promoting the progression of tumor cells. Therefore, the inhibited effects of circ_0009092 on CRC growth in vivo are might mediated in part by the lessened TAMs recruitment.

## Conclusion

In conclusion, these findings provide robust evidence that the novel circular RNA circ_0009092, which could be regulated by EIF4A3, acts as a tumor suppressor in CRC progression. Circ_0009092 inhibits tumor cell proliferation, migration, invasion, and TAMs recruitment in CRC. At the molecular level, circ_0009092 interacts with miR-665 to regulate NLK, thus exerting its biological functions in CRC. NLK regulates the phosphorylation and O-GlcNAcylation of STAT3 by binding to STAT3 and inhibits the translational expression of CCL2, thereby suppressing the TAMs infiltration in the TME. Our data suggest that circ_0009092 could be a potential biomarker and a novel therapeutic target for colorectal cancer.

## Materials and methods

### Patients and samples

Eighty paired CRC samples were obtained from patients diagnosed as CRC by pathology and received tumor radical surgery at the Zhongnan Hospital of Wuhan University. None of patients had received radiotherapy or chemotherapy before surgery. The Ethics Committee of Zhongnan Hospital of Wuhan University approved the study and informed consents were obtained from all patients.

### RNase R treatment

RNAs extracted from CRC cells were divided into two fractions. 1 µg of RNA was treated with 2 U of RNase R (Epicentre Technologies, Madison, WI, USA) or RNase-free water. The expression of circRNA_0009092 and OGDH mRNA were detected by qRT–PCR.

### Nuclear and cytoplasmic extraction

Nuclear and cytoplasm of cells were separated by Nuclear and Cytoplasmic Protein Extraction Kit (Beyotime, China, P0027) Following the manufacturer’s instructions. The RNAs of two fractions were extracted and detected by qRT–PCR. β-actin was used as cytoplasmic control, and U6 was used as nuclear control.

### Immunofluorescence (IF)

CRC cells were seeded in 6 well plate with cell-climbing slices and fixed with 4% paraformaldehyde for 30 min. Then cells were permeabilized with 0.1% TritonX-100 and incubated with 5% bovine serum albumin (BSA) for 30 min. The primary antibodies were added: anti-NLK (Cell Signaling Technology, CST, 94350, 1:100), anti-STAT3 (proteintech, 10253-2-AP, 1:200) at 4 °C overnight. Cells were incubated with anti-rabbit FITC-conjugated secondary antibody (FITC, Invitrogen, CA), Alexa-conjugated anti-goat secondary antibody (Alexa647, Invitrogen, CA), and DAPI. The cells were photographed under a fluorescence microscope.

### Immunohistochemistry (IHC)

The sections from xenograft tumor tissues and CRC patient samples were incubated with specific primary antibodies including: anti-NLK (CST, 94350,1:200), anti-CCL2 (Abcam, ab200343,1:200) at 4 °C overnight, then incubated with secondary antibodies.

### Fluorescence in situ hybridization (FISH)

Probes with 3′-Cy3 modification for circRNA_0009092 (AAGTTGGCTTCTTTAAGGTCCTGGAAGCATCTCATCAAAGC-CY5) and FAM-labeled probes specific to miR-665 (aGGGGCCTCAGCCTCCTGGt-FAM) were synthesized (Bersinbio, China), and were used for detecting the location of circRNA_0009092 and miR-665 in cells or samples according to the manufacturer’s protocols (Bersinbio, China). DAPI was used for nuclear staining.

### Luciferase gene reporter assay

The Dual-Luciferase Reporter Assay kit (Promega, USA) was used to detect the combination between circRNA_0009092 and miR-665 following the manufacturer’s instructions. Wild-type (WT) or mutant (MUT) of circRNA_0009092 were cloned into the firefly-tagged pGL3 promoter luciferase vector (Genechem, shanghai, China). CRC cells were co-transfected with circRNA_0009092 vectors or miR-665 mimic or mimic NC by Lipofectamine 2000 (Invitrogen). Luciferase activities were detected by a dual luciferase assay system (Promega, USA) after 48 h. To assess the binding between miR-665 and NLK 3’UTR, CRC cells were transfected with NLK-WT or NLK MUT vectors, and miR‐665 mimic or mimic NC by Lipofectamine 2000 (Invitrogen). To assess the STAT3 transcriptional activity, CRC cells were transfected with a CCL2 promoter luciferase vector and STAT3 vector..

### RNA immunoprecipitation (RIP) assay

The Magna RIP RNA-Binding Protein Immunoprecipitation Kit (Millipore, MA, USA) was used following the manufacturer’s protocol. 4 × 10^7^cells were lysed and then incubated with human antibody to AGO2 with protein A/G magnetic beads. Normal Mouse IgG (CST, USA) was used as negative control. The immunoprecipitation was set aside for qRT-PCR analysis.

### CoIP assay

IP/CoIP Kit (abs955, Shanghai, China) was used to perform CoIP assay. Cells were lysed and centrifuged at 4 °C, then supernatants were collected and incubated with primary antibodies overnight. Protein A and Protein G were added to the supernatants. The supernatants were then subjected to western blotting analysis. Anti-NLK (CST, USA), normal rabbit IgG (CST, USA) were used.

### Flow cytometry

Tumors of mice were cut into small pieces and digested in 1 mg/ml collagenase type IV (BioFroxx, #2091MG100, CAS 9001-12-1) and incubated at 37℃ for 1 h to obtain single-cell suspension (0.5-1×10^6^cells/ml PBS). PE-CD163, FITC-CD206, APC-CD86 (Biolegend, USA) were added in the cell and incubated for 45 min. Cells were detected on the flow cytometer. The antibodies were listed in Table S3.

### Animal models

BALB/c nude mice (female, 4‐week‐old) were purchased from Hubei Research Center of Laboratory Animals (Wuhan, China,). Animal experiment was conducted in line with the Guide for the Care and Use of Laboratory Animals of Wuhan University. The HCT-116 cells stably expressing oe-circ_0009092 or oe-NC were established. 5 × 10^6^HCT-116 cells were injected subcutaneously in BALB/c nude mice. After 8 days, THP-1 cells (10^6^) in 50μL PBS were injected into the caudal vein of mice every 3 days for seven times [39]. Tumor volumes were measured with caliper every 5 days and calculated by the formula (width2 × length)/2. The mice were euthanized after 30 days. The tumor tissues were collected for further analysis.

### Statistics analysis

Data are presented as mean ± SD of at least three independent triplicate experiments. Student’s t-test was employed to determine statistical significance between two groups. One-way ANOVA was performed for multiple comparison. The association between circ_0009092 and clinical pathology parameters were analyzed by χ2 test or Fisher exact test. Spearman correlation was used to evaluate the correlation between groups. Kaplan‐–Meier analysis was used for evaluating survival curves. Statistical analysis was performed by SPSS software version 17.0.*P*value of <0.05 was considered to be statistically significant.

### Supplementary Information

**Additional file 1**:**Figure S1.**Circ_0009092 was a single-stranded covalently closed RNA molecules. (A) The expression of circ_0009092 was detected by qRT-PCR in RNA extracts from several CRC cell lines as well as normal cell line. GAPDH was used for qRT-PCR normalization. (B) qRT-PCR analysis of the expression of circ_0009092 and OGDH mRNA after treatment with RNase R in Lovo cells. (C) The presence of circ_0009092 was validated in CRC cell lines by qRT-PCR. Divergent primers amplified circ_0009092 in cDNA but not in genomic DNA. GAPDH was used for negative control. (D) ROC curves showing predictability using circ_0009092 (AUC=0.7944). Statistical analysis between two groups was conducted using two-tailed t-test. Error bars, SD.**Figure S2.**Identification analysis of miR-665 in CRC samples. (A) FISH detection of circ_0009092 and miR-665 in CRC tumor and ANT. Nuclei were stained with DAPI. Scale bar, 50μm.**Figure S3.**Circ_0009092 inhibited CRC cell progression by targeting miR-665. (A) EdU assays were utilized to detect the viability and proliferation of Lovo and HCT-116 cells. (B) The colony formation activity of HCT116 and Lovo cells evaluated. (C) Wound healing assay were used to detect the migration of Lovo and HCT-116 cells. The migration (D) and invasion (E) ability of CRC cells were detected by transwell assays. Statistical analysis between two groups was performed using two-tailed t-test. One-way ANOVA statistical tests were adopted for more than two groups. Data are the means ± SD of three experiments. *P< 0.05, **P< 0.01, ***P< 0.001.**Figure S4.**Circ_0009092/miR-665 regulated NLK expression in CRC cells. (A) qRT-PCR analysis of the predicted target gene expression in CRC cells. GAPDH was used for qRT-PCR normalization. (B) The correlation between NLK and circ_0009092 (*n*= 80). (C) Dual-luciferase reporter assays of HCT-116 cells transfected with miR-665 mimic, NLK-WT, and NLK-MUT. Data are pooled from three independent experiments. Statistical analysis between two groups was conducted using two-tailed t-test. *P< 0.05, **P< 0.01, ***P< 0.001. ns: no significance. Error bars, SD.**Figure S5.**The expression distribution of immune score in tumor tissues and normal tissues. The abscissa represents immune cell types, and the ordinate represents the expression distribution of immune score in different groups. (A) Immune cell score heatmap, different colors represent different expression distribution in different samples. (B) The percentage abundance of tumor infiltrating immune cells in each sample. Different colors represent different types of immune cells. The abscissa represents the sample, and the ordinate represents the percentage of immune cell content in a single sample. The statistical difference of two groups was compared through the Wilcox test, significance difference of three groups was tested with Kruskal-Wallis test. *P< 0.05, **P< 0.01, ***P< 0.001.**Figure S6.**Circ_0009092/miR-665/NLK regulated CCL2 expression to affect the recruitment of macrophages. (A) Chemotaxis analysis of THP-1 macrophages with different supernatant. (B) Quantification analysis of chemotactic cells in five fields was counted manually. Statistical analysis between two groups was conducted using two‐tailed t‐test. One‐way ANOVA statistical tests were used for more than two groups. *P< 0.05, **P< 0.01, ***P< 0.001.**Figure S7.**NLK regulated the phosphorylation and O-GlcNAcylation of STAT3 by binding to STAT3. (A) Results of immunofluorescence staining showing that NLK colocalizes with STAT3 in CRC cells. (B) Interaction between STAT3 and OGT/O-GlcNAcylation modification was evaluated by coIP assays in CRC cells transfected with miR-665 mimic. (C) Interaction between STAT3 and OGT/O-GlcNAcylation modification was evaluated by coIP assays in CRC cells transfected with miR-665 inhibitor. (D) The relative CCL2 promoter transcriptional activity was detected by dual luciferase assay and normalized to Renilla luciferase activity in CRC cells transfected with oe- circ_0009092 or miR-665 mimic. (E) The relative STAT3 transcriptional activity was detected by dual luciferase assay and normalized to Renilla luciferase activity in CRC cells transfected with sh- circ_0009092 or miR-665 inhibitor. Statistical analysis between two groups was conducted using two‐tailed t‐test. One‐way ANOVA statistical tests were used for more than two groups. *P< 0.05, **P< 0.01, ***P< 0.001.**Table S1.**Clinicopathologic parameters of colorectal cancer patients (*n*= 30).**Table S2.**The sequences of the primers for qRT-PCR.**Table S3.**Antibodies used in this study.

## Data Availability

The authors declare that the data supporting the findings of this study are available within the paper, its supplementary material files

## Abbreviations

TME: Tumor microenvironment
CRC: Coloectal cancer
EMT: Epithelial-mesenchymal transition
ANT: Adjacent noncancerous tissues
IHC: Immunohistochemistry
ChIP: Chromatin immunoprecipitation
Co-IP: Co-Immunoprecipitation
