# Supplementary material for: Hsa_circ_0009092/miR-665/NLK signaling axis suppresses colorectal cancer progression via recruiting TAMs in the tumor microenvironment
Source: J Exp Clin Cancer Res. 2023 Nov 27;42:319. doi: 10.1186/s13046-023-02887-8 (PMC10680284; doi:10.1186/s13046-023-02887-8)
Supplement: Supplementary file 1 — Additional file 1:Figure S1.Circ_0009092 was a single-stranded covalently closed RNA molecules. (A) The expression of circ_0009092 was detected by qRT-PCR in RNA extracts from several CRC cell lines as well as normal cell line. GAPDH was used for qRT-PCR normalization. (B) qRT-PCR analysis of the expression of circ_0009092 and OGDH mRNA after treatment with RNase R in Lovo cells. (C) The presence of circ_0009092 was validated in CRC cell lines by qRT-PCR. Divergent primers amplified circ_0009092 in cDNA but not in genomic DNA. GAPDH was used for negative control. (D) ROC curves showing predictability using circ_0009092 (AUC=0.7944). Statistical analysis between two groups was conducted using two-tailed t-test. Error bars, SD.Figure S2.Identification analysis of miR-665 in CRC samples. (A) FISH detection of circ_0009092 and miR-665 in CRC tumor and ANT. Nuclei were stained with DAPI. Scale bar, 50μm.Figure S3.Circ_0009092 inhibited CRC cell progression by targeting miR-665. (A) EdU assays were utilized to detect the viability and proliferation of Lovo and HCT-116 cells. (B) The colony formation activity of HCT116 and Lovo cells evaluated. (C) Wound healing assay were used to detect the migration of Lovo and HCT-116 cells. The migration (D) and invasion (E) ability of CRC cells were detected by transwell assays. Statistical analysis between two groups was performed using two-tailed t-test. One-way ANOVA statistical tests were adopted for more than two groups. Data are the means ± SD of three experiments. *P< 0.05, **P< 0.01, ***P< 0.001.Figure S4.Circ_0009092/miR-665 regulated NLK expression in CRC cells. (A) qRT-PCR analysis of the predicted target gene expression in CRC cells. GAPDH was used for qRT-PCR normalization. (B) The correlation between NLK and circ_0009092 (n= 80). (C) Dual-luciferase reporter assays of HCT-116 cells transfected with miR-665 mimic, NLK-WT, and NLK-MUT. Data are pooled from three independent experiments. Statistical analysis between two gro [file 13046_2023_2887_MOESM1_ESM.docx]

Supplementary Materials for

**Hsa_****circ_0009092/miR-665/NLK signaling axis suppresses colorectal cancer progression via recruiting TAMs in the tumor microenvironment**

Jialin Song^1,2,3†^, Qing Liu^4,5†^, Lei Han ^1,2,3†^, Tiantian Song ^1,2,3†^, Sihao Huang ^1,2,3^, Xinyao Zhang^1,2,3^, Qiuming He^1,2,3^, Chenxi Liang^1,2,3^, Shuai Zhu^6*^ and Bin Xiong^1,2,3*^

+ These authors contributed equally to this work.

*** Correspondence**: Bin Xiong, Ph.D. E-mail: binxiong1961@whu.edu.cn

Shuai Zhu, Ph.D. E-mail: zhushuai@csu.edu.cn


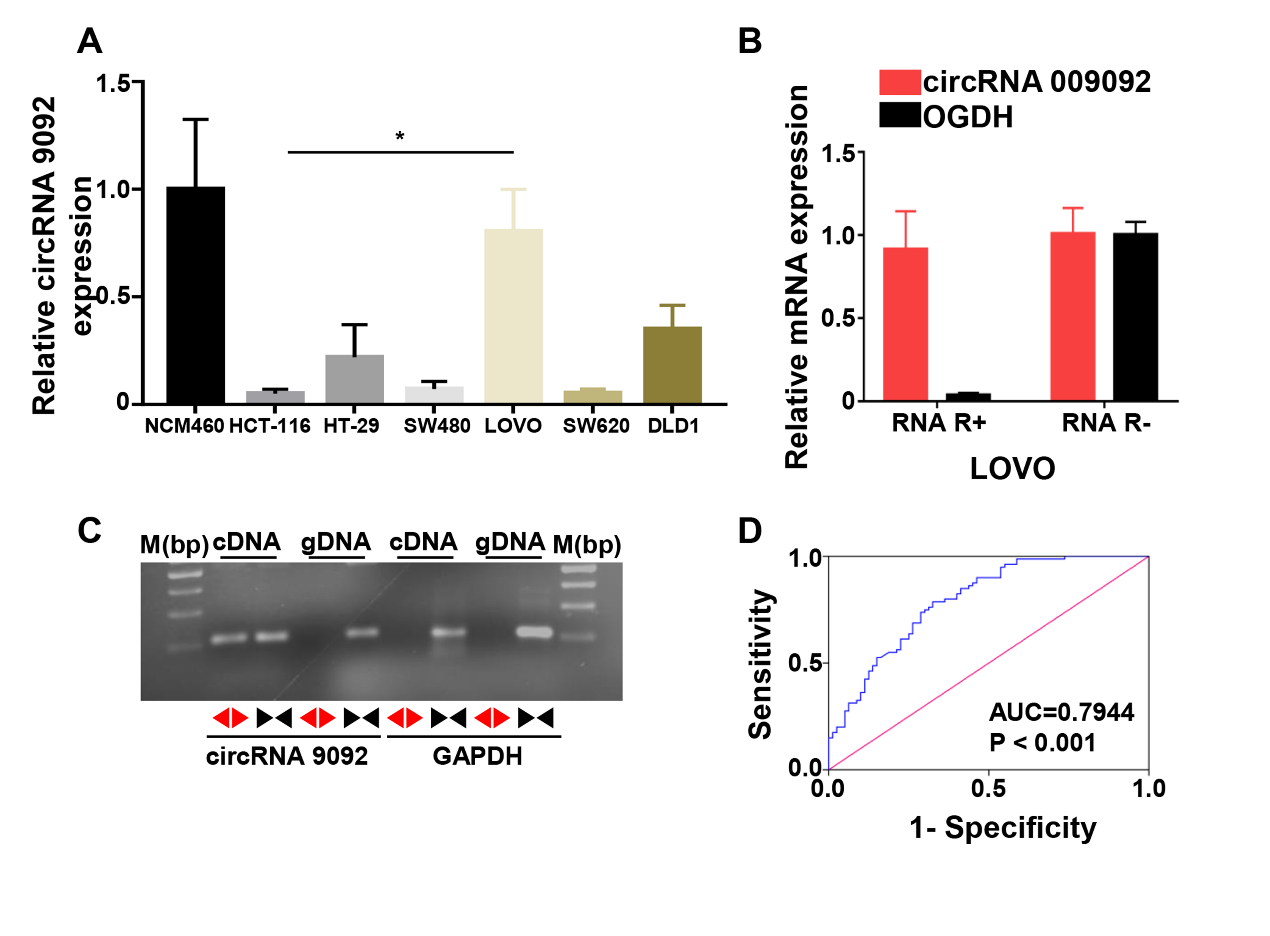


**Figure S1.** Circ_0009092 was a single-stranded covalently closed RNA molecules. **(A)** The expression of circ_0009092 was detected by qRT-PCR in RNA extracts from several CRC cell lines as well as normal cell line. GAPDH was used for qRT-PCR normalization. **(B)** qRT-PCR analysis of the expression of circ_0009092 and OGDH mRNA after treatment with RNase R in Lovo cells. **(C)** The presence of circ_0009092 was validated in CRC cell lines by qRT-PCR. Divergent primers amplified circ_0009092 in cDNA but not in genomic DNA. GAPDH was used for negative control. **(D)** ROC curves showing predictability using circ_0009092 (AUC=0.7944). Statistical analysis between two groups was conducted using two-tailed t-test. Error bars, SD.


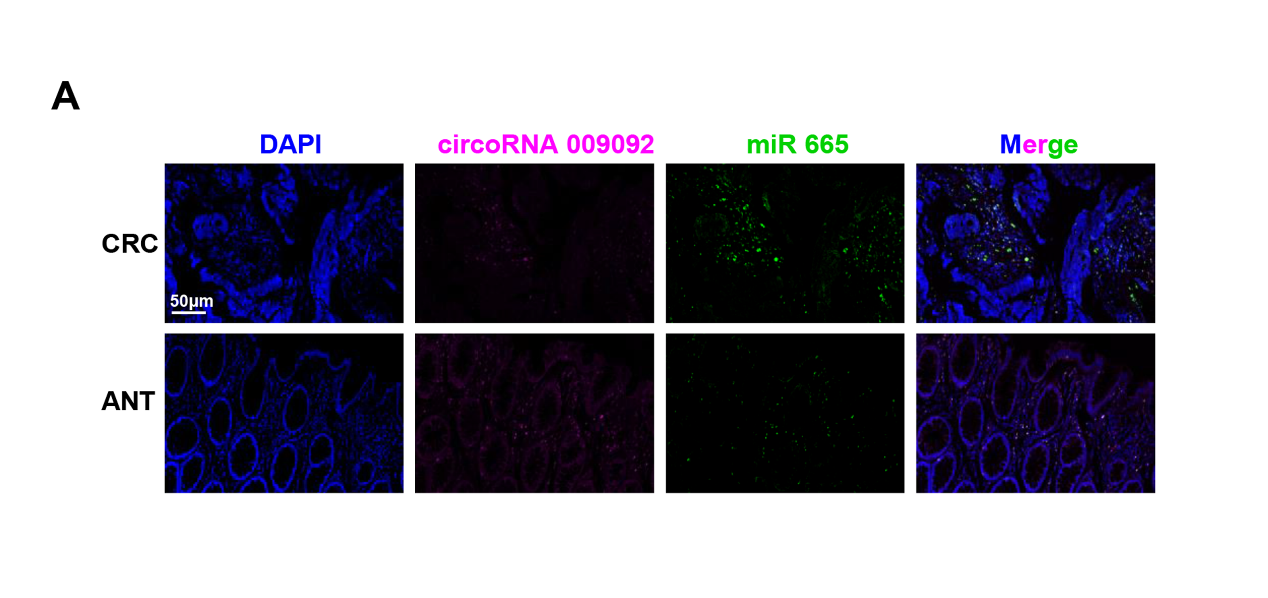


**Figure S2.** Identification analysis of miR-665 in CRC samples. **(A)** FISH detection of circ_0009092 and miR-665 in CRC tumor and ANT. Nuclei were stained with DAPI. Scale bar, 50μm.


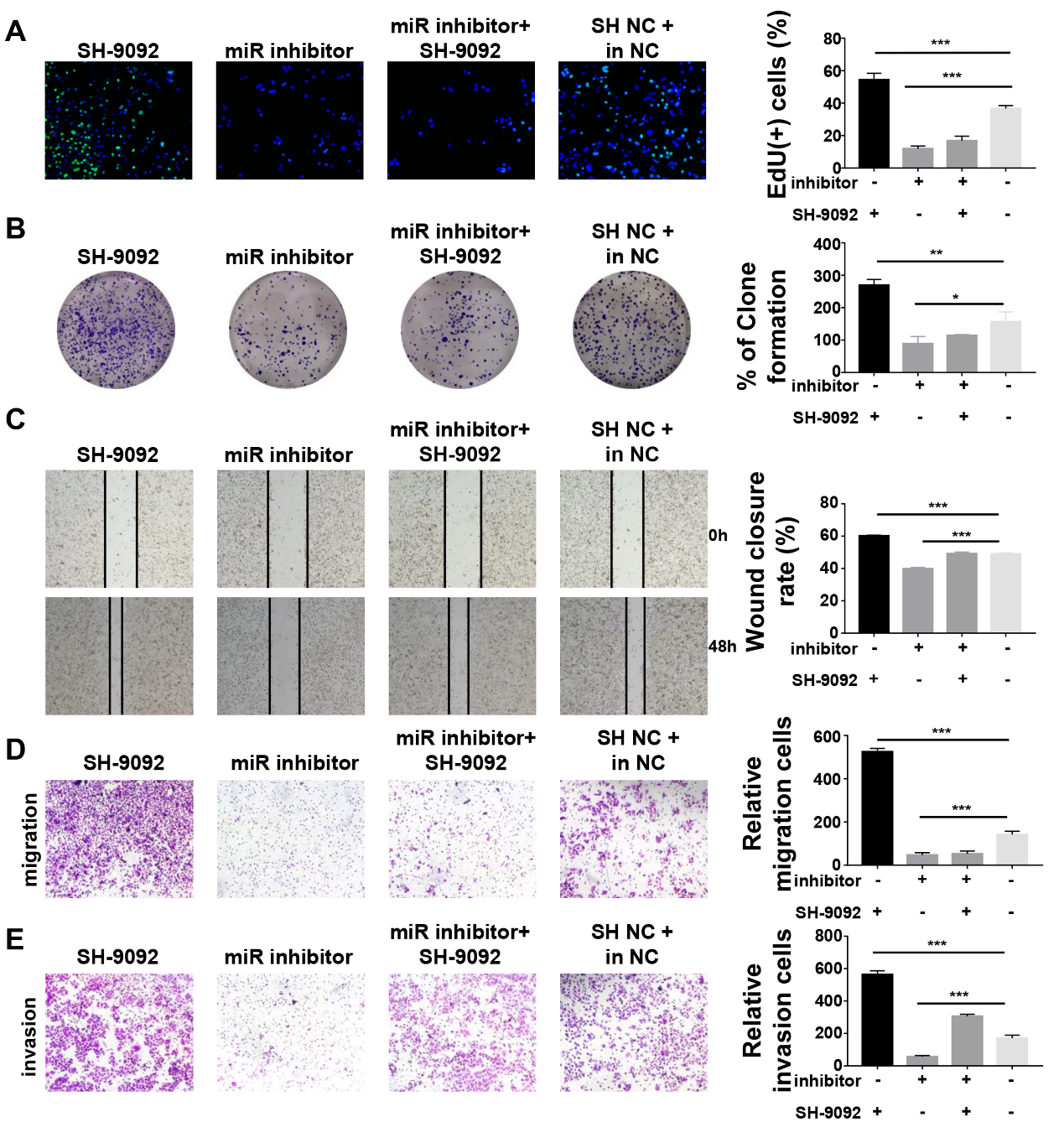


**Figure S3.** Circ_0009092 inhibited CRC cell progression by targeting miR-665. (A) EdU assays were utilized to detect the viability and proliferation of Lovo and HCT-116 cells. (B) The colony formation activity of HCT116 and Lovo cells evaluated. (C) Wound healing assay were used to detect the migration of Lovo and HCT-116 cells. The migration (D) and invasion (E) ability of CRC cells were detected by transwell assays. Statistical analysis between two groups was performed using two-tailed t-test. One-way ANOVA statistical tests were adopted for more than two groups. Data are the means ± SD of three experiments. *P < 0.05, **P < 0.01, ***P < 0.001.


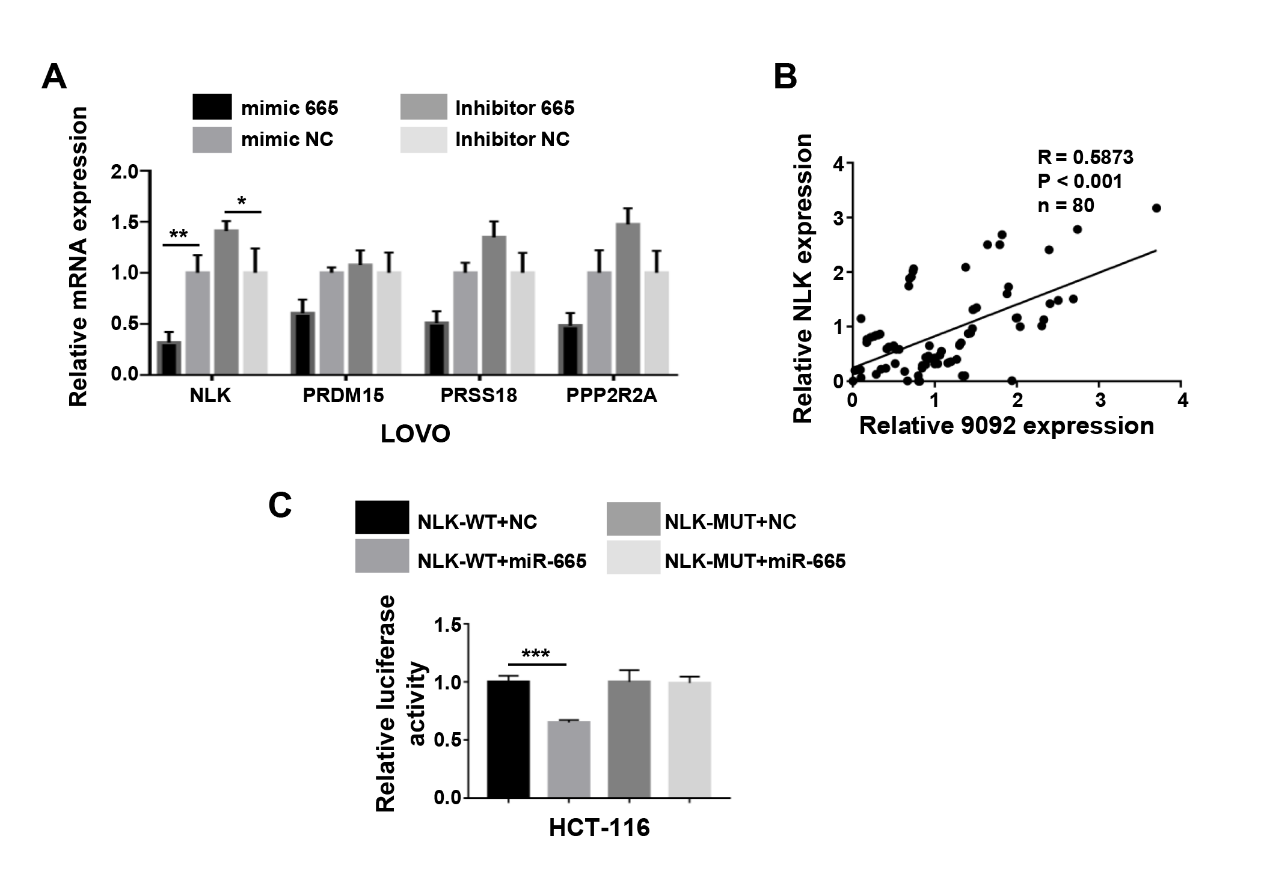


**Figure S4.** Circ_0009092/miR-665 regulated NLK expression in CRC cells. **(A)** qRT-PCR analysis of the predicted target gene expression in CRC cells. GAPDH was used for qRT-PCR normalization. **(B**) The correlation between NLK and circ_0009092 (n = 80). **(C)** Dual-luciferase reporter assays of HCT-116 cells transfected with miR-665 mimic, NLK-WT, and NLK-MUT. Data are pooled from three independent experiments. Statistical analysis between two groups was conducted using two-tailed t-test. *P < 0.05, **P < 0.01, ***P < 0.001. ns: no significance. Error bars, SD.


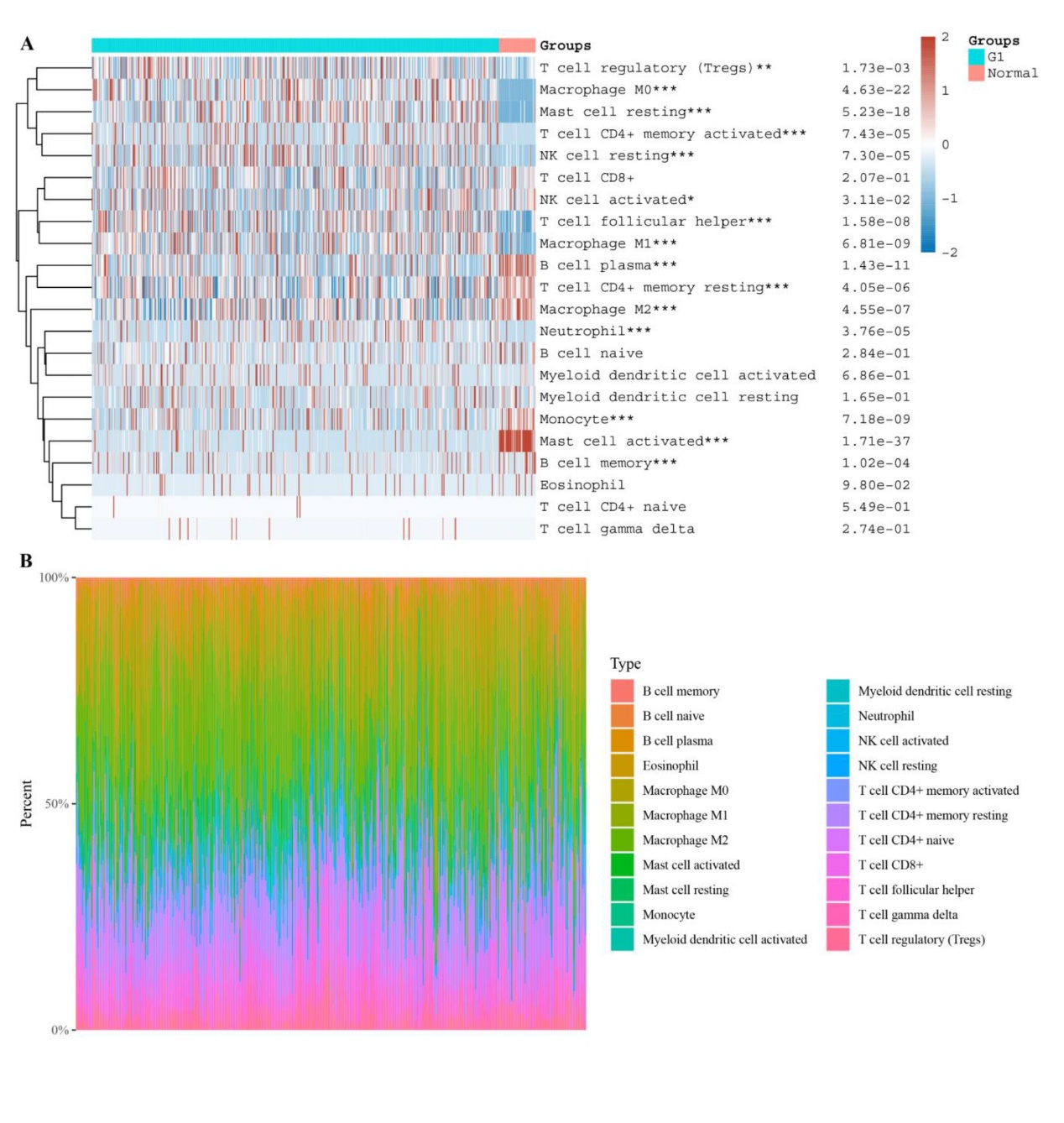


**Figure S5.** The expression distribution of immune score in tumor tissues and normal tissues. The abscissa represents immune cell types, and the ordinate represents the expression distribution of immune score in different groups. **(A)** Immune cell score heatmap, different colors represent different expression distribution in different samples. **(B)** The percentage abundance of tumor infiltrating immune cells in each sample. Different colors represent different types of immune cells. The abscissa represents the sample, and the ordinate represents the percentage of immune cell content in a single sample. The statistical difference of two groups was compared through the Wilcox test, significance difference of three groups was tested with Kruskal-Wallis test. *P < 0.05, **P < 0.01, ***P < 0.001.


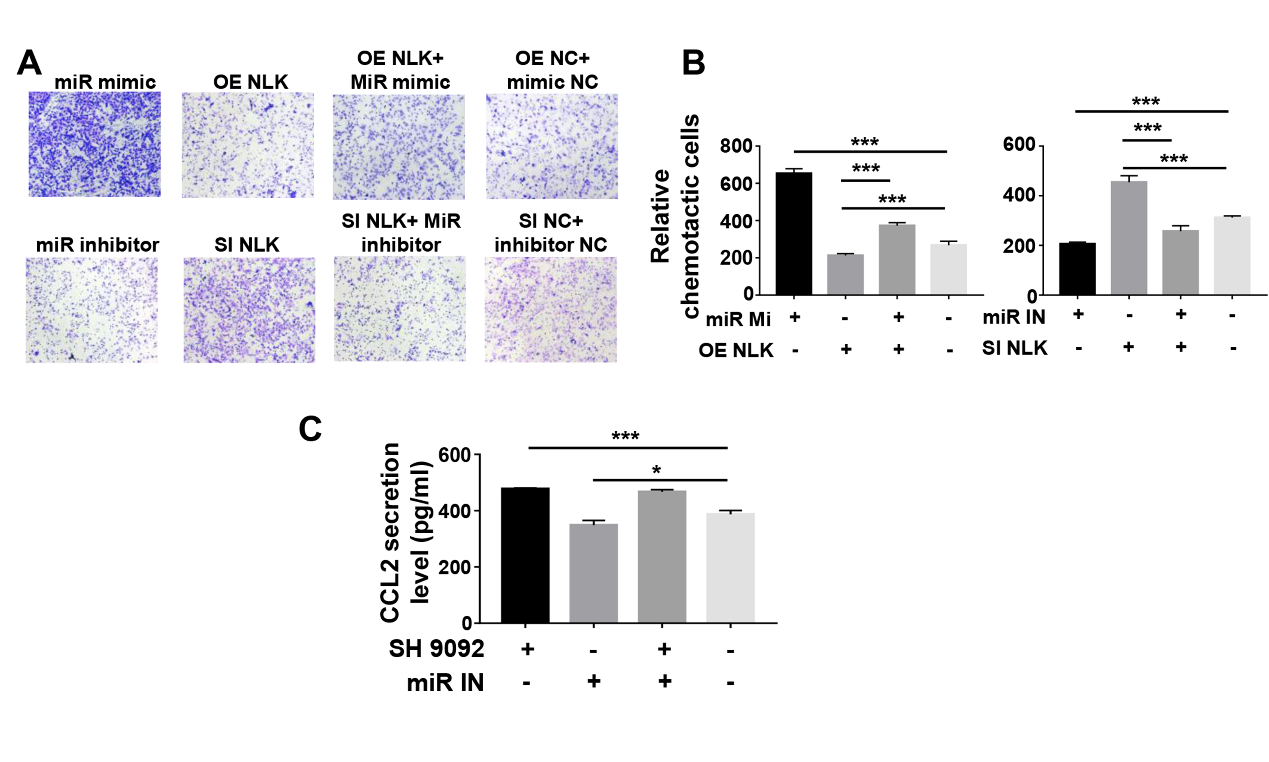


**Figure S6.** Circ_0009092/miR-665/NLK regulated CCL2 expression to affect the recruitment of macrophages. **(A)** Chemotaxis analysis of THP-1 macrophages with different supernatant. **(B)** Quantification analysis of chemotactic cells in five fields was counted manually. Statistical analysis between two groups was conducted using two‐tailed t‐test. One‐way ANOVA statistical tests were used for more than two groups. *P < 0.05, **P < 0.01, ***P < 0.001.


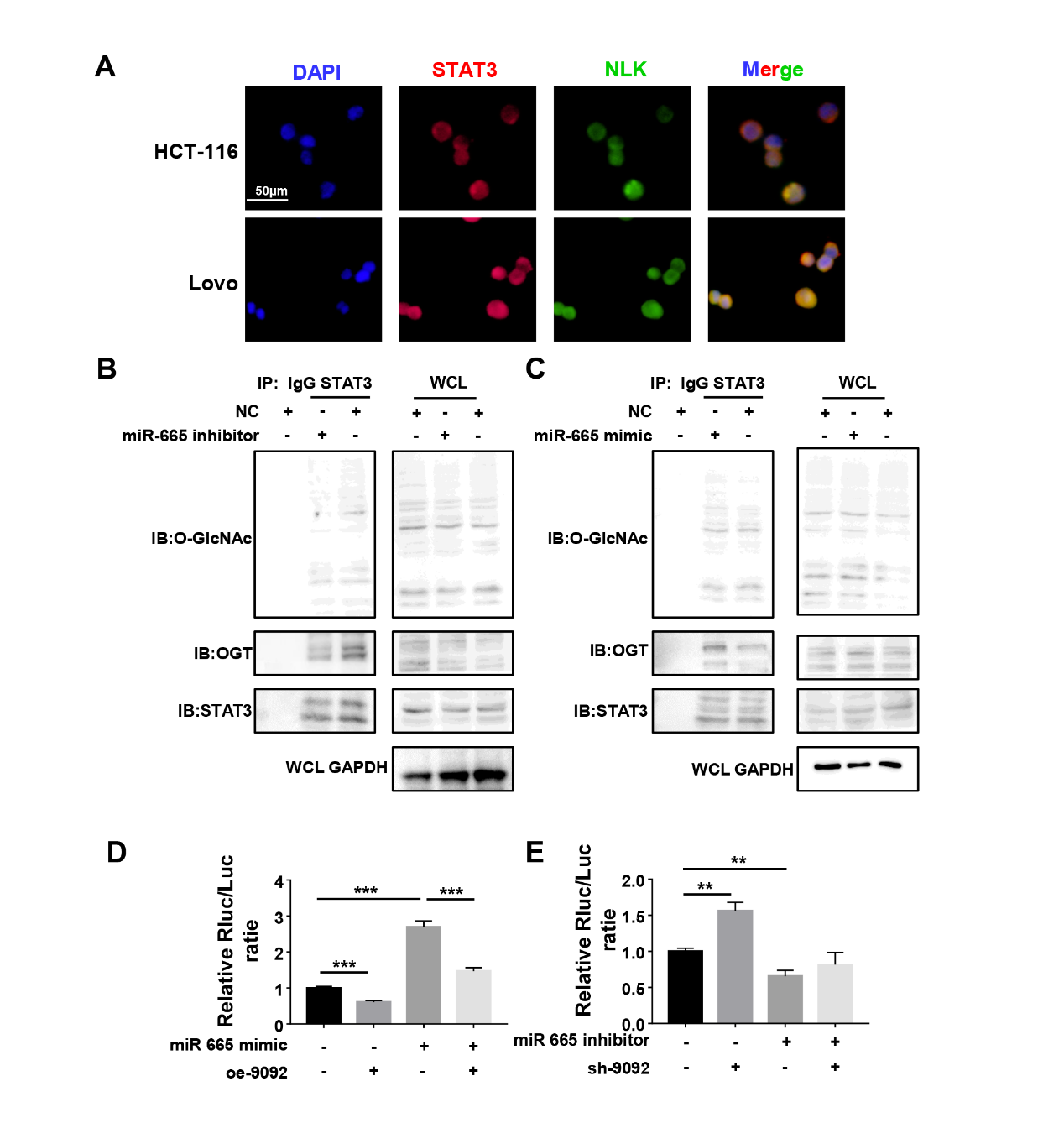


**Figure S7.** NLK regulated the phosphorylation and O-GlcNAcylation of STAT3 by binding to STAT3. **(A)** Results of immunofluorescence staining showing that NLK colocalizes with STAT3 in CRC cells. **(B)** Interaction between STAT3 and OGT/O-GlcNAcylation modification was evaluated by coIP assays in CRC cells transfected with miR-665 mimic. **(C)** Interaction between STAT3 and OGT/O-GlcNAcylation modification was evaluated by coIP assays in CRC cells transfected with miR-665 inhibitor. **(D)** The relative CCL2 promoter transcriptional activity was detected by dual luciferase assay and normalized to Renilla luciferase activity in CRC cells transfected with oe- circ_0009092 or miR-665 mimic. **(E)** The relative STAT3 transcriptional activity was detected by dual luciferase assay and normalized to Renilla luciferase activity in CRC cells transfected with sh- circ_0009092 or miR-665 inhibitor. Statistical analysis between two groups was conducted using two‐tailed t‐test. One‐way ANOVA statistical tests were used for more than two groups. *P < 0.05, **P < 0.01, ***P < 0.001.

Table S1. Clinicopathologic parameters of colorectal cancer patients (n = 30)

| Characteristic | n (%) | circ_0009092 expression | circ_0009092 expression | P value |
| --- | --- | --- | --- | --- |
|  |  | Low (n=40) | High (n=40) |  |
| Age |  |  |  | 0.682 |
| ≤60 | 28 | 13 | 15 |  |
| >60 | 52 | 23 | 28 |  |
| Gender |  |  |  | 0.264 |
| Female | 40 | 23 (28.7%) | 17 (21.2%) |  |
| male | 40 | 17 (21.2%) | 23 (28.7%) |  |
| tumor site |  |  |  | 0.176 |
| colon | 45 | 26 (32.5%) | 19 (23.8%) |  |
| rectum | 35 | 14 (17.5%) | 21 (26.2%) |  |
| tumor size (cm), median (IQR) | 80 | 4.75 (3, 5.62) | 4 (3, 7) | 0.536 |
| tumor grade |  |  |  | 0.057 |
| moderate/well | 72 | 33 (41.2%) | 39 (48.8%) |  |
| poor | 8 | 7 (8.8%) | 1 (1.2%) |  |
| LVI |  |  |  | 0.106 |
| absence | 50 | 21 (26.2%) | 29 (36.2%) |  |
| presence | 30 | 19 (23.8%) | 11 (13.8%) |  |
| PNI |  |  |  | 0.494 |
| absence | 78 | 38 (47.5%) | 40 (50%) |  |
| presence | 2 | 2 (2.5%) | 0 (0%) |  |
| T stage |  |  |  | 0.051 |
| T1+T2 | 11 | 2 (2.5%) | 9 (11.2%) |  |
| T3+T4 | 69 | 38 (47.5%) | 31 (38.8%) |  |
| N stage |  |  |  | **0.004** |
| N0 | 55 | 21 (26.2%) | 34 (42.5%) |  |
| N1+N2 | 25 | 19 (23.8%) | 6 (7.5%) |  |
| M stage |  |  |  | **0.016** |
| M0 | 62 | 26 (32.5%) | 36 (45%) |  |
| M1 | 18 | 14 (17.5%) | 4 (5%) |  |
| TNM |  |  |  | **0.002** |
| I + II | 54 | 20 (25%) | 34 (42.5%) |  |
| III + IV | 26 | 20 (25%) | 6 (7.5%) |  |

Table S2. The sequences of the primers for qRT-PCR

| Genes | Primer Sequence (5' to 3') |
| --- | --- |
| circ_0009092 | F: CATTCCGGAAGCCGTTAAT |
|  | R: CAGGAGTGGAGCAGTTGACA |
| hsa-miR-665 | Reverse Transcription (RT) primer: GTCGTATCCAGTGCAGGGTCCGAGGTATTCGCACTGGATACGACAGGGGC |
|  | F: CGCGACCAGGAGGCTGAG |
|  | R: AGTGCAGGGTCCGAGGTATT |
| NLK | F: TGAATCCCGTCATATGACTCAG |
|  | R: GATCCGTGATCAAATCCAACTG |
| EIF4A3 | F: GCTGCTTGCTCTCGGTGACTAC |
|  | R: GCTTCCTGATGTCCTCGCCAAC |
| CCL2 | F: ACCAGCAGCAAGTGTCCCAAAG |
|  | R: TTTGCTTGTCCAGGTGGTCCATG |
| U6 | F: CTCGCTTCGGCAGCACA |
|  | R: AACGCTTCACGAATTTGCGT |
| OGDH | F: ATTTTCCACGTGAACTCAGATG |
|  | R: GTAACACACCAAATCGACAACC |
| GAPDH | F: GCACCACCAACTGCTTAGCA |
|  | R: GTCTTCTGGGTGGCAGTGATG |

Table S3. Antibodies used in this study

| **Antibodies** | **Source** | **Identifier** |
| --- | --- | --- |
| NLK | Abcam | Cat# ab97642 |
| NLK | Cell Signaling Technology | Cat# 94350 |
| CCL2 | Abcam | Cat# ab200343 |
| CD68 | Abcam | Cat# ab283654 |
| p53 | Proteintech | Cat# 60283-2-Ig |
| β-catenin | Proteintech | Cat# 51067-2-AP |
| E-cadherin | Proteintech | Cat# 60335-1-Ig |
| Vimentin | Proteintech | Cat# 60330-1-Ig |
| c-myc | Proteintech | Cat# 66248-1-Ig |
| GSK-3β  p-GSK-3β | Abclonal  Abclonal | Cat# A2081  Cat# AP0039 |
| GAPDH | Proteintech | Cat# 60004-1-Ig |
| STAT3 | Proteintech | Cat# 10253-2-AP |
| p-STAT3(Tyr705) | Bioswamp | Cat# PAB36302-P |
| OGT | Proteintech | Cat#11576-2-AP |
| Anti-O-Linked N-Acetylglucosamine | Abcam | Cat#ab2739 |
| ERK1/2(137F5) | Cell Signaling Technology | Cat# 4695 |
| p-ERK1/2(Thr202/Tyr204) | Cell Signaling Technology | Cat# 4370 |
| Rabbit mAb IgG | Cell Signaling Technology | Cat# 3900 |
| Anti-Argonaute-2 | Abcam | Cat# ab186733 |
| Human CCL2/JE/MCP-1 Antibody | R&D Systems | Cat# MAB279-SP |
| PE anti-mouse CD163 Antibody | Biolegend | Cat# 156703 |
| FITC anti-mouse CD206 Antibody | Biolegend | Cat# 141703 |
| APC anti-mouse CD86 Antibody | Biolegend | Cat# 105011 |

**Materials and Methods**

**High-throughput RNA-seq**

Total RNA from 3 paired samples was quantified using the NanoDrop ND-1000. The sample preparation and microarray hybridization were performed based on the Arraystar’s standard protocols. Briefly, total RNAs were digested with Rnase R (Epicentre, Inc.) to remove linear RNAs and enrich circular RNAs. Then, the enriched circular RNAs were amplified and transcribed into fluorescent cRNA utilizing a random priming method (Arraystar Super RNA Labeling Kit; Arraystar). The labeled cRNAs were hybridized onto the Arraystar Human circRNA Array v2 (8x15K, Arraystar). After having washed the slides, the arrays were scanned by the Agilent Scanner G2505C. Agilent Feature Extraction software (version 11.0.1.1) was used to analyze acquired array images. Quantile normalization and subsequent data processing were performed using the R software limma package. Differentially expressed circRNAs with statistical significance between two groups were identified through Volcano Plot filtering. Differentially expressed circRNAs between two samples were identified through Fold Change filtering (|logFC| ≥ 2, p < 0.05). Hierarchical Clustering was performed to show the distin{Yao, 2022 #644}guishable circRNAs expression pattern among samples.

**Cell culture and Reagents**

The human monocyte cell line THP-1, human CRC cell lines (HCT116, HT-29, SW480, SW620, LOVO, DLD1), and normal cell line NCM460 were purchased from the Chinese Academy of Sciences in Shanghai. Cells were cultured in RPMI 1640 medium (Gibco, USA) with 10% fetal bovine serum (FBS) (Gibco, USA) at 37 °C in a humidified atmosphere with 5% CO2. To induce the macrophages, THP-1 cells (1×106 cells) were incubated with 100 ng/mL Phorbol 12-myristate 13-acetate (PMA, Sigma-Aldrich, USA) for 24 h. Macrophages and CRC cells co-cultivation was conducted using the non-contact co-culture transwell system (Corning, USA). Inserts containing THP-1 macrophages were transferred to 6-well plate seeded with CRC cells (1×105cells per well). After 48 h of co-culture, macrophages were harvested for further analysis.

**Quantitative real-time PCR (qRT-PCR)**

Total RNA from tissues and CRC cells were isolated using TRIzol reagent (Invitrogen, USA). RNA concentration and purity were measured using NanoDrop 2000 spectrophotometer (Thermo Fisher Scientific). RNA (1μg) was transcribed into cDNA using PrimeScript RT reagent Kit (Vazyme, China). The qRT-PCR was performed using SYBR Green master mix (Vazyme, China). β-actin, GAPDH, and U6 were used as controls, and relative mRNA and miRNA expression were calculated as 2–ΔΔCt. Primer sequences are listed in Table S2.

**Western blotting (WB)**

CRC cells were added in RIPA buffer. The proteins were separated by 10% SDS-PAGE and transferred to polyvinylidene difluoride (PVDF) membranes (Millipore, USA). Then the membranes were blocked with 5% skimmed milk for 2 h and incubated with primary antibodies at 4 °C overnight. Then the membranes were incubated with secondary antibody at room temperature for 1 h. All antibodies used in Western blot assay were shown in Table S3.

**Cell proliferation assay**

The EdU assay was performed with the BeyoClick™ EdU Cell Proliferation Kit (Beyotime, China) according to the manufacturer’s instruction and detected by using an immunofluorescence microscope. For colony formation assay, CRC cells were seeded in 6 well plate with 500 cells per well and cultured for 2 weeks. Then cells were fixed with paraformaldehyde and stained with 0.1% crystal violet.

**Wound healing assay**

CRC cells were seeded in 6 well plate and scratched with sterile pipette tip. Cells were photographed after incubation for 0 and 48 h. Image J software was used for calculating migration rate.

**Transwell migration and invasion assay**

8 μm-pore Transwell chamber (Costar, Corning, USA) was utilized for migration and invasion assay. HCT-116 and Lovo cells were seeded in the upper chamber, which were coated with (invasion) or without (migration) Matrigel and incubated for 48h. Then the migrated and invaded cells were fixed in paraformaldehyde and stained with 0.1% crystal violet. Five random fields from each well were counted under a microscope (magnification, ×200).

**Bioinformatics analysis of immune infiltration**

RNA-sequencing expression profiles and corresponding clinical information for gastric cancer samples and normal samples were downloaded from the TCGA dataset(https://portal.gdc.com). To assess the reliable results of immune score evaluation, we used immuneeconv. It’s an R software package that integrates six latest algorithms, including TIMER, xCell, MCP-counter, CIBERSORT, EPIC and quanTIseq. These algorithms had beeen benchmarked, each had a unique advantage. All the above analysis methods and R package were implemented by R foundation for statistical computing (2020) version 4.0.3 and software packages ggplot2 and pheatmap.
